# Photoresponsive Hydrogel‐Coated Upconversion Cyanobacteria Nanocapsules for Myocardial Infarction Prevention and Treatment

**DOI:** 10.1002/advs.202202920

**Published:** 2022-08-31

**Authors:** Yu Liu, Da Zhong, Yizhe He, Junkai Jiang, Weichang Xie, Zhibo Tang, Jianbin Qiu, Jun Luo, Xiaolei Wang

**Affiliations:** ^1^ Department of Rehabilitation Medicine the Second Affiliated Hospital of Nanchang University Nanchang University Nanchang 330006 China; ^2^ The National Engineering Research Center for Bioengineering Drugs and the Technologies Institute of Translational Medicine Nanchang University Nanchang 330088 China; ^3^ School of Chemistry and Chemical Engineering of Nanchang University Nanchang University Nanchang 330088 China

**Keywords:** cyanobacteria, myocardial infarction, oxygen, photoresponse

## Abstract

Myocardial infarction (MI) is a common disease that seriously threatens human health. It is noteworthy that oxygen is one of the key factors in the regulation of MI pathology procession: the controllable hypoxic microenvironment can enhance the tolerance of cardiac myocytes (CMs) and oxygen therapy regulates the immune microenvironment to repair the myocardial injury. Thus, the development of an oxygen‐controllable treatment is critically important to unify MI prevention and timely treatment. Here, a hydrogel encapsulated upconversion cyanobacterium nanocapsule for both MI prevention and treatment is successfully synthesized. The engineered cyanobacteria can consume oxygen via respiration to generate a hypoxic microenvironment, resulting in the upregulation of heat shock protein70 (HSP70), which can enhance the tolerance of CMs for MI. When necessary, under 980 nm near‐infrared (NIR) irradiation, the system releases photosynthetic oxygen through upconversion luminescence (UCL) to inhibit macrophage M1 polarization, and downregulates pro‐inflammatory cytokines IL‐6 and tumor necrosis factor‐*α* (TNF‐*α*), thereby repairing myocardial injury. To sum up, a photoresponsive upconversion cyanobacterium nanocapsule is developed, which can achieve MI prevention and treatment for only one injection via NIR‐defined respiration and photosynthesis.

## Graphical abstract

The hydrogel encapsulated upconversion cyanobacteria nanocapsules are developed for the prevention and treatment of acute myocardial infarction by using a flexible NIR defined in vivo oxygen modulating, through respiration and photoresponsive photosynthesis of cyanobacteria.

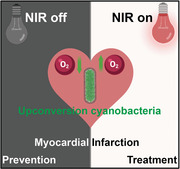

## 1 Introduction

Cardiovascular disease associated with myocardial infarction (MI) is a major cause of morbidity and mortality worldwide.^[^ ^1^ ^]^ Due to the neglect of preventive measures for MI high‐risk populations, the reduction of cardiac myocytes (CMs) caused by long‐term and repeated ischemia results in difficult‐to‐recover injury and scar tissue.^[^ ^2^ ^]^ The widely used treatments like drug and surgical treatments cannot reverse the massive loss of functional CMs after MI, which highlights the importance of the combination of MI prevention and treatment.^[^ ^3^ ^]^ Ischemic preconditioning, a method of MI prevention, has been confirmed to have a certain therapeutic effect on MI.^[^ ^4^ ^]^ The mechanism of prevention is that after one or more times of ischemia‐reperfusion stimulations in a short period of time, hypoxia and ischemia in the myocardial microenvironment will upregulate the expression of protective substances to improve the tolerance of CMs. Ischemia‐reperfusion induced directly through the circulatory system might cause cardiovascular adverse events to a great extent with uncontrollable location, extent, and time.^[^ ^5^ ^]^ For MI treatment, oxygen therapy remains the routine method, which improves cardiac function and regulates the immune microenvironment.^[^ ^6^ ^]^ However, many studies have confirmed that high oxygen fraction caused by excessive oxygen therapy may lead to vasoconstriction, an increase in vascular resistance, and a decrease of myocardial oxygen supply, thereby aggravating MI injury.^[^ ^7^ ^]^ Thus, an accurate oxygen‐modulating treatment shows significant importance for MI prevention and therapy.

Recent progress has been made in injectable hydrogels for myocardial protection, and some of the hydrogels have entered the stage of clinical trials.^[^ ^8^ ^]^ Hydrogels as carriers have been always used to deliver cells,^[^ ^9^ ^]^ bioactive molecules,^[^ ^10^ ^]^ drugs,^[^ ^11^ ^]^ and nanoparticles.^[^ ^12^ ^]^ Although injectable hydrogels are promising as a kind of clinical treatment for MI, controlled oxygen‐modulating hydrogel for MI prevention and therapy has not been developed. *Synechococcus elongatus* (*S. elongatus*) PCC 7942 (cyanobacteria), a freshwater unicellular organism, liberates oxygen through photosynthesis.^[^ ^13^ ^]^ Unlike other gram‐negative bacteria, the structurally distinct lipopolysaccharide (LPS) on its surface does not cause a strong inflammatory response, which makes it a potential oxygen source in vivo.^[^ ^14^ ^]^ With its photosynthesis and good biocompatibility, some biomedical applications have been developed, such as tumor photodynamic therapy,^[^ ^15^ ^]^ treatment of diabetic wounds,^[^ ^16^ ^]^ and so on.^[^ ^17^ ^]^ But most of these researches ignored the consumption of oxygen by respiration of cyanobacteria in dark environments, which might achieve photoresponsive treatment for MI combined with oxygen liberation through photosynthesis.

**Figure 1:**
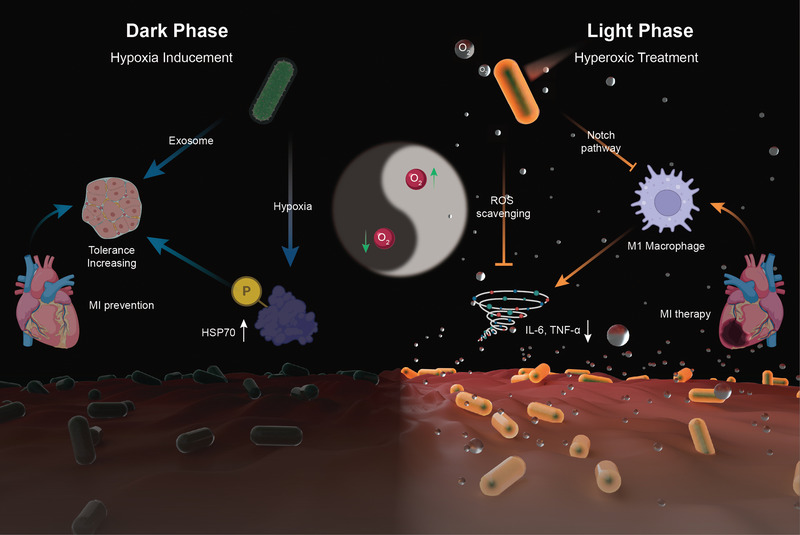
The schematic illustration for the synthesis of UCCy@Gel for acute MI prevention (dark phase) and therapy (light phase): the UCCy@Gel consumes oxygen via respiration to generate a hypoxic microenvironment, resulting in the upregulation of HSP70, which enhanced the tolerance of CMs for MI (left); Under 980 nm NIR irradiation, the UCCy@Gel releases photosynthetic oxygen through UCL to inhibit macrophage M1 polarization and downregulated proinflammatory cytokines IL‐6 and TNF‐*α* to repair myocardial injury (right).

Herein, we reported a hydrogel‐coated upconversion cyanobacterium nanocapsule (UCCy@Gel) for MI prevention and therapy (**Figure** **1**): upconversion nanoparticles (*β*‐NaErF_4_@NaLuF_4_ nanocrystals, UCNPs), which could absorb deeper tissue penetrable near‐infrared (NIR) photons and emit shorter wavelength photons (upconversion luminescence, UCL),^[^ ^18^ ^]^ were conjugated to the surface of cyanobacteria. Besides, a methacrylate outer hydrogel layer was formed to enhance the stability and provided stable tissue adhesion. The upregulation of heat shock protein70 (HSP70) following the controllable local hypoxic microenvironment caused by the respiration of cyanobacteria in the dark inhibited the expression of the apoptosis protein cysteinyl aspartate specific proteinase‐3 (Caspase‐3) to prevent MI. When necessary, through the excitation of 980 nm NIR light, a blend of visible light emitted by UCNPs was used for photosynthesis in cyanobacteria to achieve appropriate oxygen liberation. By this means, a flexible NIR‐controllable oxygen‐modulating cyanobacterium nanocapsule could be realized for both MI hypoxic prevention and local oxygen therapy.

## 2 Result and Discussion

### 2.1 Synthesis and Characterization of UCNPs and UCCy@Gel

**Figure 2:**
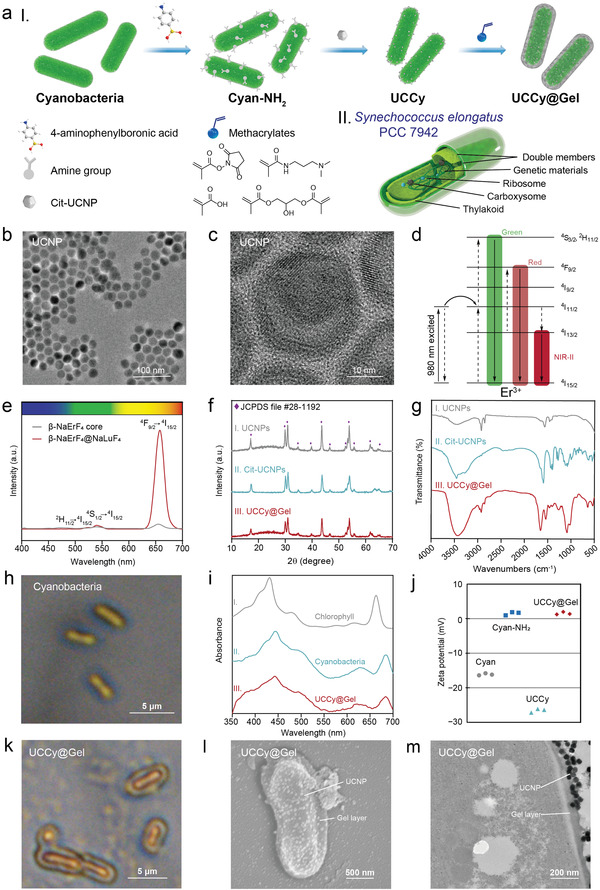
Preparation and characterization of UCCy@Gel. a) I‐Schematic illustration for the synthesis of UCCy@Gel, II‐the schematic illustration for the structure of cyanobacteria b) TEM image of UCNPs. Scale bar =  100 nm. c) HRTEM image of UCNPs. Scale bar =  10 nm. d) The UCL mechanism of UCNPs. e) The UCL spectra of UCNPs. f) The XRD analysis of I‐UCNPs, II‐Cit‐UCNPs, and III‐UCCy@Gel. g) The Fourier transform infrared spectroscopy (FTIR) spectra of I‐UCNPs, II‐Cit‐UCNPs, and III‐UCCy@Gel. h) Photograph of cyanobacteria under an optical microscope. Scale bar =  5 µm. i) UV–vis spectra of I‐chlorophyll, II‐cyanobacteria, and III‐UCCy@Gel. j) Zeta potentials of cyanobacteria, Cyan‐NH_2_, UCCy, and UCCy@Gel. k) Photograph of UCCy@Gel under an optical microscope. Scale bar =  5 µm. l) SEM image of UCCy@Gel. Scale bar =  400 nm. m) TEM image of UCCy@Gel. Scale bar =  200 nm.

The schematic illustration for the synthesis of UCCy@Gel was shown in **Figure** **2**a‐I. First, cyanobacteria were modified with amino groups to bind UCNPs to obtain upconversion bacteria (UCCy). Then, the methacrylate hydrogel layer was formed by free‐radical copolymerization, resulting in UCCy@Gel.

UCNPs were synthesized using the high‐temperature coprecipitation method.^[^ ^20^ ^]^ Transmission electron microscopy (TEM) and high‐resolution TEM (HRTEM) images of UCNPs were shown in Figure 2b,c, respectively. The particle size of UCNPs was about 28 nm, out of which 14 nm was the hexagonal phase (*β*) NaErF_4_ nanocrystals core, and with an average shell thickness of about 7 nm (Figure S1, Supporting Information). Erbium ions (Er^3+^) can offer multiple excitations and emission pathways spanning the visible to the NIR wavelengths, which attributes to its rich energy level system.^[^ ^19^ ^]^ The up‐converted green and red emission peaks at 520, 550, and 650 nm were attributed to the transitions from ^2^H_11/2_, ^4^S_3/2_, and ^4^F_9/2_ of Er^3+^, respectively, and UCL was simultaneously enhanced with a thick interfacial NaLuF_4_ layer because it eliminated surface quenching (Figure 2d,e).^[^ ^21^ ^]^ Powder X‐ray diffraction (XRD) analysis shown in Figure 2f‐I further confirmed the crystallinity and hexagonal phase of the synthesized core‐shell nanocrystals.

Since as‐synthesized UCNPs were covered by hydrophobic oleic acid ligands (OA‐UCNPs), a ligand exchange was required. To endow UCNPs with hydrophilicity, UCNPs were treated with nitrosonium tetrafluoroborate and sodium citrate using a ligand exchange method with slight modifications.^[^ ^22^ ^]^ Ligand exchange from OA‐UCNPs to Cit‐UCNPs was confirmed by the infrared absorption peaks located at 1580, 1417, and 1390 cm^−1^ (Figure 2f and Figure S2, Supporting Information). Powder XRD analysis shown in Figure 2f‐II also verified the successful preparation of Cit‐UCNPs.

*S. elongatus* PCC 7942 was routinely cultivated under photoautotrophic conditions with continuous illumination at 28 °C,^[^ ^23^ ^]^ which could be observed under an optical microscope and scanning electron microscopy (SEM) (Figure 2h and Figure S3, Supporting Information). It possesses a double membrane structure and is regarded as a gram‐negative bacterium. Genetic materials, ribosomes, carboxysomes, and thylakoids are contained within the membrane (Figure 2a‐II).^[^ ^24^ ^]^ Cyanobacteria contain chlorophyll within their thylakoids, which absorb light energy and give cyanobacteria their green color. To characterize the absorption properties, we analyzed cyanobacteria and cyanobacterial chlorophyll by UV–vis spectroscopy. The two major absorption peaks were located at 440 and 665 nm, which corresponded to the dominant absorption of blue and red light, respectively (Figure 2i‐I).

To improve the ligation efficiency, cyanobacteria were first modified with amino groups to generate amino‐modified cyanobacterial (Cyan‐NH_2_). The polysaccharides on the surface of cyanobacteria provided natural active sites for the conjugation of boric acid. 4‐aminophenylboronic acid hydrochloride was used as an aminating reagent because the boron center could coordinate with vicinal hydroxyl groups of polysaccharides to yield boronate esters at a fast rate.^[^ ^25^ ^]^ The change of zeta potential demonstrated the success of aminating reaction (Figure 2j).

The ligation of cyanobacteria and Cit‐UCNPs was carried out using an amidation reaction. Carboxyl groups on the surface of Cit‐UCNPs were activated by introducing a widely used water‐soluble condensation agent 1‐ethyl‐3‐(3‐dimethylaminopropyl) carbodiimide, and then reacted with Cyan‐NH_2_ to obtain UCCy. From the SEM image, it could be observed that the UCNPs were successfully attached to the surface of the cyanobacteria (Figure S4, Supporting Information). The zeta potential of UCCy changed due to unreacted carboxyl groups of Cit‐UCNPs (Figure 2j).

UCCy@Gel was fabricated via copolymerization of methacrylate on the surface of UCCy. N‐succinimidyl methacrylate (NMS) was synthesized and characterized by ^1^H nuclear magnetic resonance (^1^H NMR) (Figure S5, Supporting Information). First, UCCy was modified with methacryloyl groups by reacting with NMS, then the copolymerization was triggered by ammonium persulfate and N,N,N″,N″‐tetramethylethylenediamine. Finally, 4‐aminophenylboronic acid hydrochloride was added and then react with the hydrogel‐encapsulated UCCy to obtain UCCy@Gel. NMS and methacrylic acid (MA) residues were introduced in the hydrogel coating of UCCy in consideration of the adhesion of tissues.^[^ ^26^ ^]^ The phenylboronic structure of 4‐aminophenylboronic acid hydrochloride could scavenge reactive oxygen species (ROS). The hydrogel layer could be observed under an optical microscope (Figure 2k). SEM and TEM images of UCCy@Gel were shown in Figure 2k,l, respectively. UCCy@Gel exhibited a near‐neutral zeta potential of about 1.5 mV, while the zeta potentials for cyanobacteria and UCCy were approximately −16.1 and −26.6 mV, respectively (Figure 2j). The diffraction peaks of UCNPs from XRD analysis (Figure 2f‐III) and the characteristic peaks at 2935 cm^−1^ (alkyl C−H stretching) 1402 cm^−1^ (C−N stretching), 1278 cm^−1^ (C−O stretching) and the peaks of the carbonyl group at 1663 cm^−1^ were due to C=O stretching was shown in FTIR spectra (Figure 2g‐III) further indicated the successful synthesis of UCCy@Gel. UV–vis spectrum of UCCy@Gel showed only slight changes in absorption properties of UCCy@Gel throughout the synthesis process (Figure 2i).

### 2.2 UCL property of UCCy@Gel

**Figure 3:**
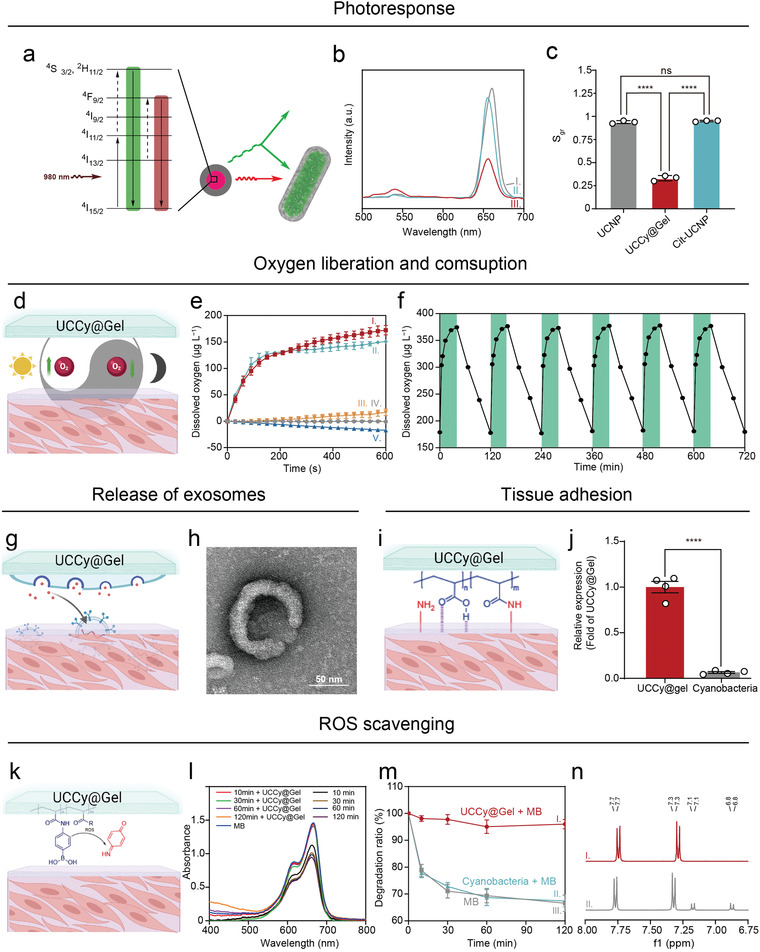
The functional characterization of UCCy@Gel. a) The possible energy conversion upon excitation of NIR light irradiation. b) The UCL spectra of I‐UCNPs, II‐Cit‐UCNPs, and III‐UCCy@Gel. c) The quantitative spectral purity analysis of upconversion red light. d) The schematic diagram of photosynthesis and respiration of UCCy@Gel. e) Time‐oxygen liberation curves of I‐UCCy@Gel + 980 nm NIR light irradiation, II‐UCCy@Gel + white light irradiation, III‐Cyanobacteria + 980 nm NIR light irradiation, IV‐PBS + 980 nm NIR light irradiation, and V‐UCCy@Gel in dark. f) The activity of UCCy@Gel (green = NIR on; white = dark treatment). g) The schematic diagram of exosomes from UCCy@Gel. h) TEM image of exosomes from UCCy@Gel. i) The schematic diagram of UCCy@Gel bonds tissues. j) The quantitative data of UCCy@Gel bonds tissues. k) The schematic diagram of ROS scavenging. l) The UV–vis spectra of MB under different conditions at different times. m) The MB degradation ratio of I‐UCCy@Gel + MB, II‐cyanobacteria + MB, III‐MB. n) ^1^H NMR spectra of I‐before oxidation, II‐after oxidation. *p < 0.05, **p < 0.01, ***p < 0.001, ****p < 0.0001. Data are means ± SD (*n* ≥ 3).

The possible energy conversion upon excitation of NIR light irradiation was shown in **Figure** **3**a. We analyzed luminescent spectra of UCNPs of UCNPs, Cit‐UCNPs, and UCCy@Gel (Figure 3b). The green/red spectral purity of UCL, *S*_gr_, were quantified using the equation:

(1) $$S_{gr}=\frac{A_{r}-A_{g}}{A_{r}+A_{g}}$$

where *A*_g_ and *A*_r_ are the integrated areas under the emission peak of green light and emission peak of red light, respectively.^[^ ^27^ ^]^ The difference between UCNPs and Cit‐UCNPs was not statistically significant while there was a significantly reduced *S*_gr_ of UCCy@Gel, which could be considered as a phenomenon caused by the absorption of light by cyanobacteria (Figure 3c).

### 2.3 Oxygen Liberation of UCCy@Gel

The schematic diagram of photosynthesis and respiration of UCCy@Gel is shown in Figure 3d. We analyzed oxygen liberation from UCCy@Gel via a portable dissolved oxygen meter. UCCy@Gel under NIR light (980 nm light‐emitting diode) showed higher photosynthesis activity than that under natural white light.^[^ ^28^ ^]^ Due to the respiration of UCCy@Gel in the dark, the dissolved oxygen concentration in the solution decreased by 600 s (≈ 17.08 µм). Upon exposure to the NIR light, UCCy@Gel (1 × 10^6^ cells mL^−1^) produced a high amount of oxygen within 600 s at a level of about 172 µм in time‐dependent manners (Figure 3e), with a higher rate than that observed in white light (≈ 150.8 µм).

Next, we confirmed the activity of UCCy@Gel by using NIR light irradiation or dark treatment and monitored the concentration of dissolved oxygen. Under NIR light irradiation, the dissolved oxygen concentration of the UCCy@Gel (1 × 10^6^ cells mL^−1^) increased gradually to about 380 µм in 30 min; under dark conditions, the dissolved oxygen decreased from about 380 µм to 180 µм in 90 min (Figure 3d). These results demonstrated that the cyanobacteria in the UCCy@Gel still had high activity and could carry out photosynthesis and respiration. The experimental device was shown in Figure S6, Supporting Information.

An oxygen probe [Ru(dpp)_3_]Cl_2_ was used to validate the oxygen generation in UCCy@Gel (Figure S7, Supporting Information). [Ru(dpp)_3_]Cl_2_ is a luminescent probe that is commonly used for oxygen indication and quantification. Molecular oxygen could induce a significant decrease in the luminescence of [Ru(dpp)_3_]Cl_2_ due to dynamic quenching. The decrease of red fluorescence after 5 min irradiation confirmed the generation of oxygen. Extracellular vesicles (EVs), released as part of the normal physiology of cells, can be broadly divided into two categories, ectosomes and exosomes. Exosomes were isolated from the culture medium of UCCy@Gel, which have been proven to have the potential for healing tissues (Figure 3g,h).^[^ ^29^ ^]^

### 2.4 The Tissue Bonding and ROS Scavenging Ability of UCCy@Gel

NMS and MA residues for the hydrogel layer of UCCy@Gel could interact with tissues via hydrogen bonds and amidation reactions, which provided non‐covalent and covalent binding (Figure 3i).^[^ ^26^ ^]^ The bond between UCCy@Gel and tissues was measured by co‐incubation with fresh mice hearts. After co‐incubation for 1 h, the free UCCy@Gel were quantified by measuring the absorbance at 730 nm of supernatant (Figure 3j).^[^ ^30^ ^]^ Compared to cyanobacteria, UCCy@Gel showed much stronger tissue adhesion, which suggested that the NMS and MA residues interact with tissues to enhance the binding force.

ROS are a group of active molecules and free radicals. Overexpression of ROS leads to oxidative stress, which can damage cellular proteins, DNA, and lipids. Excessive ROS production after MI can eventually lead to heart failure. Thus, it is necessary to eliminate ROS in the early occurrence of MI. The reaction between peroxides and organic boric acid is widely known.^[^ ^31^ ^]^ Due to the responsiveness of phenylboronic structure to oxidative stimuli, phenylboronic acid was used as a ROS scavenging group (Figure 3k).^[^ ^32^ ^]^ Methylene blue (MB), a redox reporter molecule that was degraded under oxidative conditions, was used as a probe molecule. Fenton's reagent, that is, ferrous salt and H_2_O_2_, is a common reagent to produce hydroxyl radicals. After incubation with Fenton's reagent, the color of the MB solution rapidly turned from dark blue to almost colorless (Figure S8, Supporting Information). In the presence of Fenton's reagent, the absorbance of MB in the control group was significantly reduced compared with that of the UCCy@Gel group (Figure 3l). By establishing a standard curve for MB (Figure S9, Supporting Information), the degradation ratio of MB was quantified (Figure 3m). Compared to the control group, the rapid quenching of the dichlorodihydrofluorescein probe suggested a fast scavenging rate of ROS (Figure S10, Supporting Information).

We studied the mechanism of scavenging ROS and identified the final product of the oxidation reaction as 4‐iminocyclohexa‐2,5‐dien‐1‐one by ^1^H NMR (Figure 3n). The absorption peak of the charge‐transfer complex consisted of an electron‐rich aromatic ring in the system (phenolic compounds, intermediates) and an electron‐deficient aromatic ring (4‐iminocyclohexa‐2,5‐dien‐1‐one) was observed by UV–vis spectroscopy (Figure S11, Supporting Information). The possible process of oxidation was that phenylboronic acid was first converted to phenolic compounds and further oxidized to give the final product.

### 2.5 In Vitro Cell Protection Experiments of UCCy@Gel

**Figure 4:**
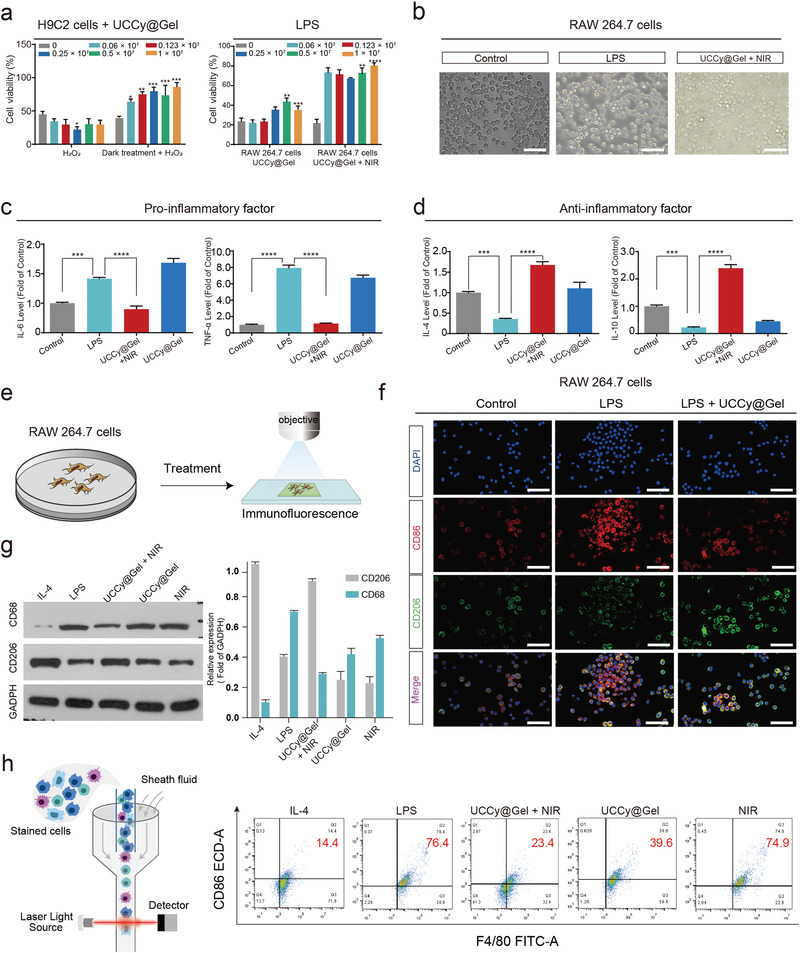
The functional validation of UCCy@Gel on cells. a) In vitro, H_2_O_2_ and LPS were used to induce cardiomyocytes (H9C2) and macrophages (RAW 264.7) damage, respectively, as well as verified the resistance of UCCy@Gel to H_2_O_2_ damage following hypoxia treatment with H9C2, and the role of oxygen release in inhibiting LPS‐induced RAW 264.7 damage. b) Macrophage polarization states of each group under light microscopy. Scale bar = 200 µm. c,d) Expression levels of pro‐inflammatory (IL‐6 and TNF‐*α*) and anti‐inflammatory factors (IL‐4 and IL‐10) in each group were assayed by enzyme‐linked immunosorbent assay (ELISA). Control: PBS treated; LPS: LPS (50 ng mL^−1^); UCCy@Gel (1 × 10^6^ cells mL^−1^) + 980 nm NIR irradiation; UCCy@Gel (1 × 10^6^ cells mL^−1^). e,f) Fluorescence microscopy demonstrated differential surface expression of macrophage M1 and M2 phenotypic markers. Diamidinyl phenyl indole (DAPI, nuclei) = blue, CD86 (M1) = red, CD206 (M2) = green. Scale bar =  200 µm. g) Western blot analysis revealed the presence of CD68 and CD206 expression. h) Flow cytometry results showed the surface expression of CD86. *p < 0.05, **p < 0.01, ***p < 0.001, ****p < 0.0001. Data are means ± SEM (*n* ≥ 3).

We first evaluated the biocompatibility of UCCy@Gel in three cell lines by calcein acetoxymethyl ester (Calcein‐AM)/propidium iodide (PI) staining assay, including mouse macrophage leukemia cells (RAW 264.7), human umbilical vein endothelial cells (HUVECs) and mouse fibroblasts (L929) (Figures S12–S14, Supporting Information), and the negligible positive PI staining illustrated the excellent biosafety of UCCy@Gel. To further confirm the protection of CMs and oxygen immunoregulation under light irradiation of UCCy@Gel, cardiac myoblast cells (H9C2) and RAW 264.7 were used in an in vitro hydrogen peroxide (H_2_O_2_) and LPS‐induced injure model, respectively.^[^ ^33^ ^]^ After co‐incubation with UCCy@Gel for 24 h, the enhanced tolerance to H_2_O_2_ injury of CMs was testified by cell counting kit‐8 (CCK‐8) assay. Further western blot analysis showed the upregulation of HSP70 (Figure S15, Supporting Information) and the inhibition of CMs apoptosis induced by H_2_O_2_ (Figure S16, Supporting Information). Here, we first induced the macrophages into the M1 type by co‐incubating with LPS for 12 h, then added UCCy@Gel and exposed to NIR light irradiation for 20 min, and finally identified the cell type after replacing the supernatant for 24 h. The enhancement of H9C2 resistance against H_2_O_2_ and macrophage resistance against LPS‐induced inflammatory injury was shown in **Figure** **4**a, respectively. We also found that oxygen generated under light irradiation suppressed macrophage polarization to the M1 state under a microscope (Figure 4b), reduced the levels of pro‐inflammatory (TNF‐*α* and IL‐6), and increased the levels of anti‐inflammatory cytokines (Figure 4c,d). The decrease in the cluster of differentiation 86 (CD86), a pro‐inflammatory marker, was observed in M1 polarized macrophages, and upregulation of cluster of differentiation 206 (CD206), an M2 macrophage marker, was detected by fluorescence microscopy imaging (Figure 4e,f).^[^ ^34^ ^]^ The same trend was observed in western blot and flow cytometry experiments (Figure 4g,h). The results above all indicated that coincubation with UCCy@Gel in the dark (hypoxic pre‐treatment) could enhance the tolerance of CMs, and oxygen generated under light irradiation efficiently suppressed M1 macrophage polarization, reduced anti‐inflammatory cytokines released, and regulated the immune microenvironment, which were similar to results in previous literature.^[^ ^35^ ^]^

### 2.6 UCCy@Gel Enhances MI Tolerance

**Figure 5:**
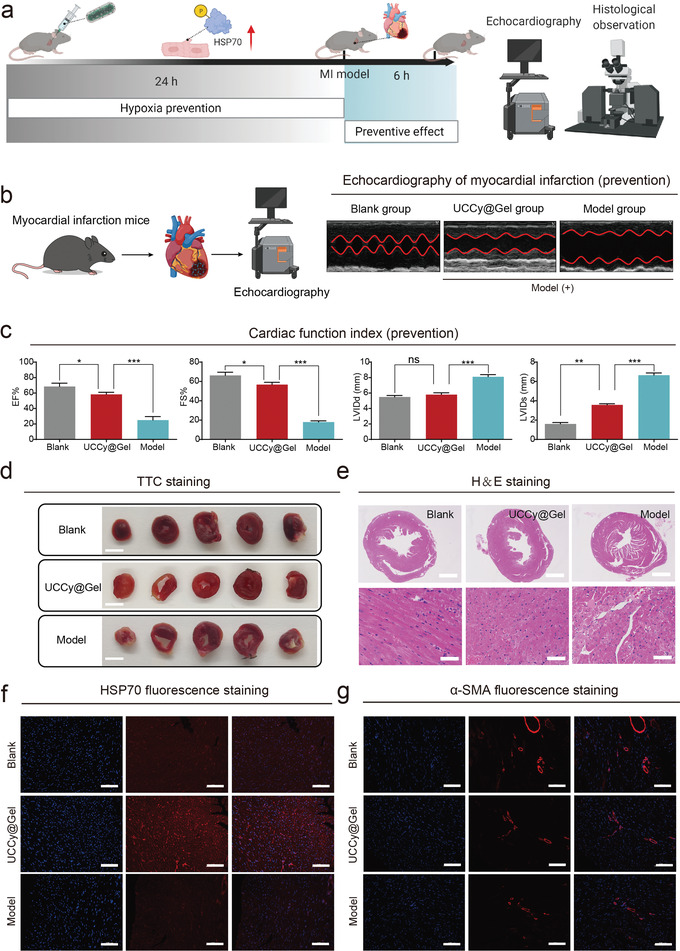
The cardioprotective function of UCCy@Gel in vivo. a) The schematic diagram to verify the protective effect of UCCy@Gel. b) Echocardiographic examination of mouse hearts. c) Heart function was evaluated by echocardiography. EF: ejection fraction; FS: fractional shortening; LVIDd: left ventricular internal diameter at end‐diastole; LVIDs: left ventricular internal diameter at end‐systole. d) TTC staining, Scale bar = 5 mm. e) The H&E staining observation of mouse heart. Scale bar = 1 mm. Scale bar = 50 µm. f) The heat shock HSP70 protein fluorescent staining observation. Scale bar = 100 µm. g) The fluorescence staining of *α*‐SMA protein. Scale bar = 100 µm. *p < 0.05, **p < 0.01, ***p < 0.001, ****p < 0.0001. Data are means ± SEM (*n* ≥ 3).

An apical ultrasound‐guided injection was performed to transplant UCCy@Gel into the cardiac apex to validate the enhanced tolerance of CMs after dark treatment. With the help of a small animal imaging system, we observed that UCCy@Gel could be stably retained within 7 days (Figure S17, Supporting Information), and the apical injection of UCCy@Gel did not cause damage to the heart in 30 days (Figure S18, Supporting Information). As shown in **Figure** **5**a, we first injected UCCy@Gel into the cardiac apex of the heart and then performed MI modeling after 24 h of dark treatment. After 6 h of modeling, mice cardiac function was monitored by echocardiography (ejection fraction = EF%; fractional shortening = FS%; left ventricular internal diameter at end‐diastole = LVIDd; left ventricular internal diameter at end‐systole = LVIDs).^[^ ^36^ ^]^ It was found that UCCy@Gel dark hypoxic pre‐treatment could reduce the decline of cardiac function caused by MI, which played a certain role in maintaining cardiac function (Figure 5b,c). Subsequent triphenyltetrazolium chloride (TTC) staining, hematoxylin and eosin (H&E) staining, and Masson staining of mice hearts also demonstrated a certain degree of cardiac tissue protection (Figure 5d,e and Figure S19, Supporting Information). It was worth noting that clinical and biomedical research revealed that HSP70 overexpression was involved in the cardioprotection.^[^ ^37^ ^]^ To explore the mechanism of the UCCy@Gel protection, we stained the heart tissue of mice treated with different groups for HSP70, and the results showed that UCCy@Gel dark hypoxia pretreatment could promote HSP70 protein expression (Figure 5f). Thus, the upregulation of HSP70 protein in CMs induced by hypoxic pretreatment might be the key mechanism for its cardioprotection. Subsequent, fluorescence staining of *α*‐smooth muscle actin (*α*‐SMA), an important marker of cardiac fibrosis, also showed that UCCy@Gel dark hypoxia pretreatment could reduce its expression (Figure 5g). In addition, the same trend was observed in wheat germ agglutinin (WGA) and sirius red staining (Figures S20 and S21, Supporting Information).

### 2.7 UCCy@Gel Regulates the Immune Microenvironment for MI Treatment

**Figure 6:**
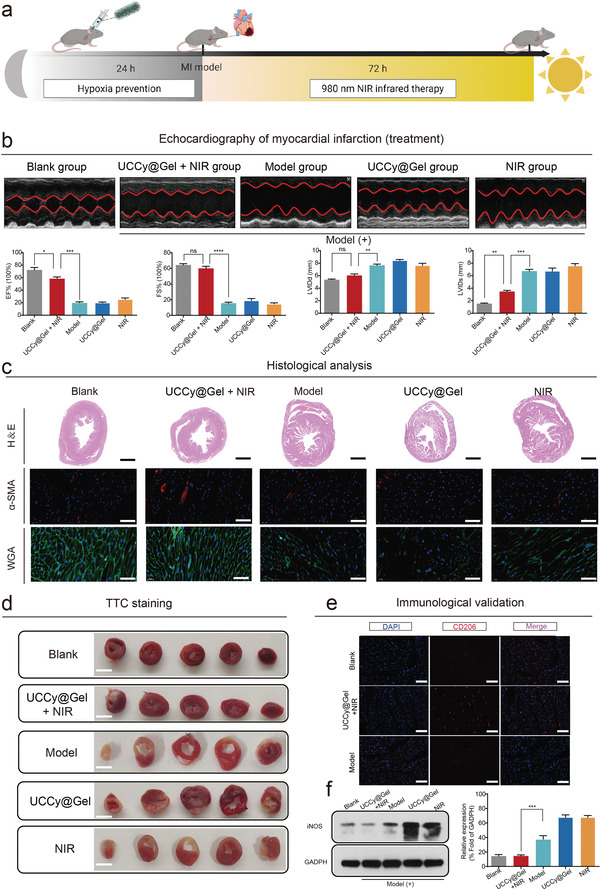
The cardiac therapeutic function of UCCy@Gel in vivo. a) Schematic of MI treatment: mice were first injected with UCCy@Gel and treated in a dark environment for 24 h before MI was modeled, and finally, MI mice were treated with 980 nm NIR irradiation for 72 h (in a homemade NIR cage). b) Heart function was evaluated by echocardiography. EF: ejection fraction; FS: fractional shortening; LVIDd: left ventricular internal diameter at end‐diastole; LVIDs: left ventricular internal diameter at end‐systole. c) Heart H&E staining, *α*‐SMA staining, and WGA staining. H&E, Scale bar = 1 mm. *α*‐SMA and WGA, Scale bar = 50 µm. d) TTC staining, Scale bar = 5 mm. e) CD206 immunofluorescence staining of mice hearts. DAPI (nuclei) = blue, CD206 (M2) = red. Scale bar = 100 µm. f) Western blot and quantitative analysis of iNOS (M1 macrophage marker). *p < 0.05, **p < 0.01, ***p < 0.001, ****p < 0.0001. Data are means ± SEM (*n* ≥ 3).

To verify that UCCy@Gel could regulate the immune microenvironment and downregulate the inflammatory factors after MI caused by photosynthetic oxygen, we first injected mice with UCCy@Gel and treated them in a dark environment for 24 h before MI was modeled. Finally, the MI mice were treated with 980 nm NIR (in a homemade NIR cage) for 72 h (**Figure** **6**a and Figure S22, Supporting Information). We examined heart function by echocardiography (EF%; FS%; LVIDd; LVIDs) after the end of treatment. The result showed that UCCy@Gel could improve MI‐induced cardiac dysfunction (Figure 6b). The H&E, *α*‐SMA, WGA, TTC, and Masson staining showed similar results (Figure 6c,d and Figure S23, Supporting Information). The immune microenvironment was further evaluated by immunofluorescence staining of the mouse heart, which revealed the upregulation of CD206 (Figure 6e) and downregulation of CD86 (Figure S24, Supporting Information). In addition, we also demonstrated that UCCy@Gel had anti‐inflammatory effects and decreased expression of proinflammatory cytokines such as IL‐6 and TNF‐*α* (Figure S25, Supporting Information). Finally, we performed western blot experiments on cardiac tissue, demonstrating the downregulation of inducible nitric oxide synthase (iNOS) protein, which indicated inhibition of macrophage polarization to the M1 state and modulation of the immune microenvironment to reduce myocardial injury (Figure 6f). Vital organs, such as the liver and kidney, were harvested for histopathologic examination and no significant pathological changes were found in the organs of all groups (Figures S26–S28, Supporting Information).

### 2.8 The Mechanism Exploration of Hypoxic Prevention and Oxygen Therapy

**Figure 7:**
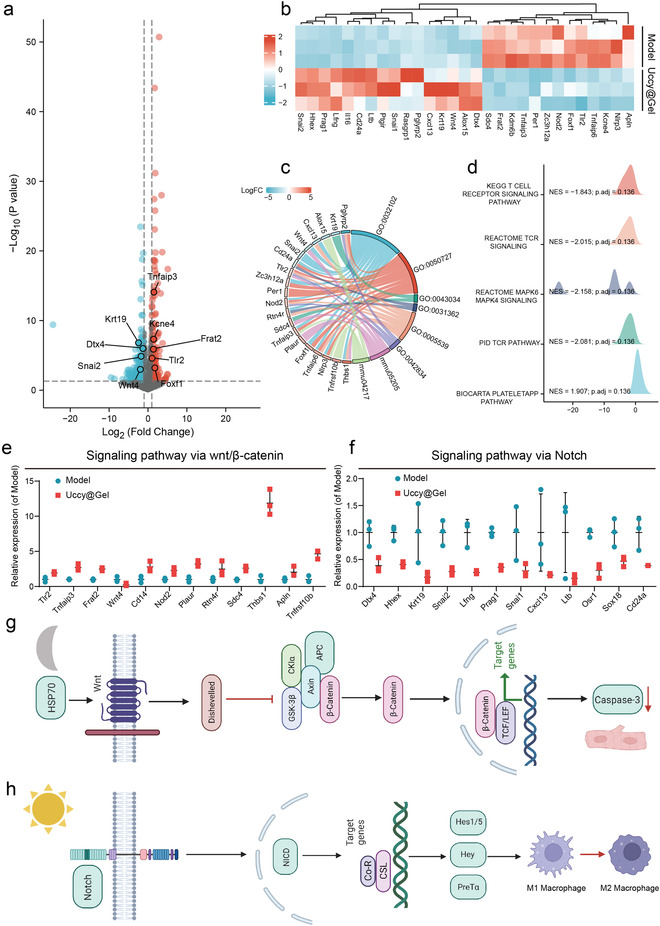
The UCCy@Gel mechanism of action. a) Volcano plot of differentially expressed genes determined by whole‐transcriptome RNA‐seq (gray: genes that are not significantly different; red: upregulated genes; blue: downregulated genes). b) Heatmap of differentially expressed genes. (red indicating relatively high expressed genes, blue indicating relatively low expressed genes). c) GO differential gene analysis. d) KEGG visual network data analysis. E,f) Analysis of differential gene expression of Wnt/*β*‐catenin and Notch pathway. g) The upregulation of HSP70 activated the Wnt/*β*‐catenin pathway and downregulates caspase‐3 in CMs after dark treatment. h) Activation of the Notch pathway suppressed M1 macrophages polarization after oxygen therapy. Data are means ± SEM (*n* ≥ 3).

As described above, the therapeutic effects on MI of UCCy@Gel mainly included the following two aspects: The hypoxic microenvironment caused by the respiration of UCCy@Gel could upregulate HSP70 of CMs before MI. Besides, the controllable oxygen generation via photosynthesis could regulate the immune microenvironment, thereby reducing the heart damage after MI. To explore the molecular mechanism, we performed transcriptomic sequencing to analyze differential gene expression of the MI modeling group and UCCy@Gel treatment group using *DESeq2*. The resulting volcano plot was shown in **Figure** **7**a. The 30 highest fold‐change genes after UCCy@Gel treatment were illustrated in a heat map (Figure 7b). We performed pathway enrichment analysis against the Kyoto Encyclopedia of Genes and Genomes (KEGG) pathway (Figure 7c,d), and the results displayed noticeable changes in several inflammatory pathways in multiple signaling. During the related inflammatory pathways, the myocardial protection effect of the Wnt/*β*‐catenin pathway has been extensively studied in recent years.^[^ ^38^ ^]^ We, therefore, analyzed differential gene expression in the Wnt/*β*‐catenin pathway (Figure 7e). Emerging evidence indicates that the Notch signaling pathway plays an important role in the regulation of macrophage polarization.^[^ ^39^ ^]^ Hence, we focused on analyzing the expression of Notch pathway differential genes (Figure 7f). Based on these results, we hypothesized that the upregulation of HSP70 activated the Wnt/*β*‐catenin pathway to downregulate caspase‐3 in dark; oxygen therapy activated the Notch pathway to suppress macrophage polarization to M1 type (Figure 7g,h).

## 3 Conclusion

In conclusion, we designed NIR‐responsive hydrogel‐coated upconversion cyanobacteria nanocapsules (UCCy@Gel) for MI prevention and treatment. The engineered cyanobacteria system could consume oxygen by respiration to generate a controllable local hypoxic microenvironment, which could upregulate HSP70 to enhance the tolerance of CMs. To reverse the inflammatory immune microenvironment for myocardial repairing, UCCy@Gel could respond to 980 nm NIR irradiation to achieve continuous and controllable oxygen generation after MI. Transcriptomic sequencing was performed to analyze the mechanism of hypoxic prevention and oxygen therapy in vivo. In addition, with the good in vivo biocompatibility, a long retention time of UCCy@Gel in myocardial tissue was validated by using a small animal imaging system, which supported its significant controllable minimally invasive therapy was suitable for future clinical applications. However, there are still some shortcomings of this work: 1) apical injection is an invasive modality, and controllable minimally invasive is an important follow‐up research direction. 2) We just measured the effectiveness of UCCy@Gel in a 4‐day in vivo experiment before and after MI, and its long‐term effects remain to be verified. 3) Although deeper tissue penetrable NIR light (980 nm) was used, the application in larger animals still needs to be further explored due to limited penetration depth. 4) The metabolism and long‐term biosafety of UCCy@Gel as well as the changes in the immune system still need further exploration. 5) We only suggested a possible mechanism of action, which has not been further verified in‐depth using molecular biology methods. In summary, we achieved hypoxic prevention and oxygen therapy by only once injection using both the respiration and photosynthesis of membrane‐engineered cyanobacteria, which provided an interesting and feasible protocol for photosynthesis to achieve accurate oxygen modulating in vivo. Since the importance of oxygen to tissues (and organs) is self‐evident, we believe this strategy can also have scientific significance for other ischemic diseases such as stroke and ischemic nephropathy.

## 4 Experimental Section

### Ethical Statement

All animal experiments were performed under the guidelines of the National Institutes of Health and approved by the Animal Ethics Committee of Nanchang University, Nanchang, China (SYXK 2018‐0006).

### Statistical Analysis

All data were expressed as mean ± SEM or SD. Data was analyzed using Prism 8.0 (GraphPad Software), one‐way analysis of variance, and Student's *t*‐test were used for statistical analysis. The *p‐*value < 0.05 was considered statistically significant.

Other experimental details are reported in the Supporting Information.

## Conflict of Interest

The authors declare no conflict of interest.

## Supporting information

Supporting Information

## Data Availability

The data that support the findings of this study are available from the corresponding author upon reasonable request.
