# Supplementary material for: Photoresponsive Hydrogel‐Coated Upconversion Cyanobacteria Nanocapsules for Myocardial Infarction Prevention and Treatment
Source: Adv Sci (Weinh). 2022 Aug 31;9(30):2202920. doi: 10.1002/advs.202202920 (PMC9596827; doi:10.1002/advs.202202920)
Supplement: Supplementary file 1 — Supporting Information [file ADVS-9-2202920-s001.pdf]

ADVANCED SCIENCE
Open Access

Supporting Information

for *Adv. Sci.*, DOI 10.1002/advs.202202920

Photoresponsive Hydrogel-Coated Upconversion Cyanobacteria Nanocapsules for Myocardial Infarction Prevention and Treatment

Yu Liu, Da Zhong, Yizhe He, Junkai Jiang, Weichang Xie, Zhibo Tang, Jianbin Qiu, Jun Luo and Xiaolei Wang**

Supporting Information

Photoresponsive Hydrogel-Coated Upconversion Cyanobacteria Nanocapsules for Myocardial Infarction Prevention and Treatment

Yu Liu, Da Zhong, Yizhe He, Junkai Jiang, Weichang Xie, Zhibo Tang, Jianbin Qiu, Jun Luo and Xiaolei Wang**

Supporting Information

Materials

Erbium (III) acetate hydrate, Lutetium (III) acetate hydrate, 1-octadecene and oleic acid were purchased from Sigma-Aldrich. Ammonium fluoride was purchased from Alfa-Aesar. Sodium citrate, N-(3-(dimethylamino)propyl)methacrylamide (DMAPMA), glycerol dimethacrylate (GDA), 4-aminophenylboronic acid hydrochloride, nitrosyl tetrafluoroborate, isopropanol, hexane, cyclohexane, N-hydroxysuccinimide (NHS), 1-(3-Dimethylaminopropyl)-3-ethylcarbodiimide hydrochloride, tetrahydrofuran (THF), ethanol, N,N-dimethylformamide (DMF) and sodium hydroxide were all purchased from Shanghai Aladdin Bio-Chem Technology Co., LTD. Potassium carbonate, ammonium persulfate (APS), N,N,N',N'-tetramethylethylenediamine (TEMED), methacrylic acid (MA) and Tris(4,7-diphenyl-1,10-phenanthroline)ruthenium (II) dichloride complex were purchased from Shanghai Macklin Biochemical Co., Ltd. Ethyl acetate, petroleum ether and 3 Å molecular sieves were purchased from Sinopharm Chemical Reagent Co., Ltd. BG-11 medium was purchased from Qingdao Hope Bio-Technology Co., Ltd. All other reagents were purchased from commercial resources and used as received unless otherwise noted.

Characterization

Analytical thin-layer chromatography (TLC) was performed using E. Merck silica gel 60 F254 precoated plates (0.25 mm). The developed chromatogram was analyzed by ultraviolet (UV) lamp (254 nm). Nuclear magnetic resonance (NMR) spectra were recorded on Bruker Advance III (400 MHz) spectrometers with tetramethylsilane as an internal standard. The morphology of the as-synthesized nanoparticles was observed by field emission transmission electron microscopes (TEM, Tecnai G2 20, Thermo, US) and field emission scanning electron microscopy (SEM, ZEISS Sigma 300, England). Fourier transform infrared (FTIR) spectroscopy was performed on Nicolet iS20 (Thermo, US). Zeta potential was measured by Z90s (Malvern, England). X-ray diffraction (XRD) pattern was recorded by a D8A A25 X (Bruker, Germany). The fluorescence spectra were measured by a fluorescence spectrophotometer (FL970, Techcomp, China). The ultraviolet–visible (UV–vis) absorption data were collected by

UV-2600 spectrophotometer (SHIMADZU, Japan). The stained cells were analyzed using microscope (Olympus, Japan) and inverted fluorescence microscopy (Olympus, Japan). The absorbance of cytotoxicity was measured by the microplate reader of VICTOR Nivo 3S (PerkinElmer, UK). The *in vivo* fluorescence imaging was recorded by IVIS Lumina XR III (Perkin Elmer).

Experiment Section

Synthesis of N-succinimidyl methacrylate (NMS)

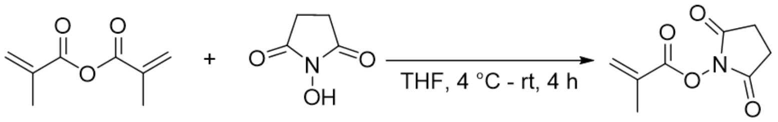

A solution of methacrylic anhydride (1.54 g, 10 mmol) in dry tetrahydrofuran (THF, 10 mL) was added to a stirred mixture of N-hydroxysuccinimide (1.38 g, 12 mmol), potassium carbonate (1.66 g, 12 mmol) and THF (15 mL) at 4 °C. Then the reaction mixture was allowed warm to room temperature and stirred for 4 h. After the solvent was evaporated in vacuo, deionized (DI) water (50 mL) was added. The mixture was extracted by ethyl acetate (50 mL). The organic phase was dried over sodium sulfate anhydrate. The mixture was then filtered and the solvent was evaporated in vacuo to obtain the product.

^{1}H NMR (400 MHz, $CDCl_3$) δ = 6.42 (s, 1H; CH), 5.89 (s, 1H; CH), 2.86 (s, 4H; CH_2), 2.06 (s, 3H; CH_3).

Synthesis of β-$NaErF_4$@$NaLuF_4$ (UCNPs)

1. Synthesis of hexagonal phase (β) $NaErF_4$ core

Erbium acetate tetrahydrate (833 mg, 2 mmol) was added to a mixture of oleic acid (9 mL) and 1-octadecene (30 mL) in a 100 mL Schlenk flask under argon atmosphere. The mixture was heated to 120 °C in vacuo for 30 min to remove water. A clear solution was obtained after water removed. The solution was cooled down to room temperature, and a methanolic solution containing ammonium fluoride (296 mg, 8 mmol) and sodium hydrate (200 mg, 5 mmol) was added. The mixture was stirred for about 1 h, and methanol was removed by heating the mixture to 140 °C under argon flow. Then the mixture was heated to 300 °C rapidly (10 °C per min) and maintained for 60 min to obtain the β-$NaErF_4$ nanocrystals. After the solution was cooled down, ethanol (60 mL) was added to precipitate the nanocrystals. The crude product was finally obtained by centrifugation at 11000 rpm. The product was washed with ethanol for several times

and dispersed in cyclohexane (20 mL).

2. Synthesis of UCNPs

Lutetium acetate hydrate (833 mg, 2 mmol) was added to a mixture of oleic acid (18 mL) and 1-octadecene (16 mL) in a 100 mL Schlenk flask under argon atmosphere. The mixture was heated to 120 °C in vacuo for 30 min to remove water. A clear solution was obtained after water was removed. Then β-$NaErF_4$ core (20 mL, 100 mM in 1-octadecene) was added to the solution. The mixture was heated to 120 °C under argon flow for 30 min to remove cyclohexane. The solution was cooled down to room temperature, and a methanolic solution containing ammonium fluoride (296 mg, 8 mmol) and sodium hydrate (200 mg, 5 mmol) was added. The mixture was stirred for about 1 h, and methanol was removed by heating the mixture to 140 °C under argon flow. Then the mixture was heated to 300 °C rapidly (10 °C per min) and maintained for 60 min to obtain the β-$NaErF_4$@$NaLuF_4$ nanocrystals. After the solution was cooled down, ethanol (40 mL) was added to precipitate the nanocrystals. The crude product was finally obtained by centrifugation at 11000 rpm. The product was washed with ethanol for several times and dispersed in cyclohexane (20 mL).

To enhance the luminescence efficiency of nanocrystals, the inert core was thicken using another 2 mmol of Lu (III) complex to obtain UCNPs.

Synthesis of Cit-UCNPs

The ligand change of UCNPs was based on the procedures reported in the literature.[1] 5 mL of UCNPs dispersed in hexane (5 mg mL^{-1}) was combined with 5 mL of DMF solution of $NOBF_4$ (0.01 M). The resulting mixture was shaken gently. After about 5 min, 10 mL isopropanol was added to precipitate nanocrystals. After centrifugation to remove the supernatant, the precipitated nanocrystals were washed and redispersed in DI water (5 mL).

Sodium citrate (25 mg) was added to above solution and the reaction mixture was sonicated for 60 min. Another 5 mL of isopropanol was added to precipitate nanocrystals. Cit-UCNPs were collected by centrifugation and washed with DI water.

Synthesis of UCCy@Gel

1. Amino-modification of *Synechococcus elongatus* PCC 7942 (cyanobacteria)

Briefly, a solution of 4-aminophenylboronic acid hydrochloride (2.5 mg mL^{-1} in phosphate buffer saline (PBS) buffer) and cyanobacteria were mixed in the dark for 4 h. The resulting mixture was purified by centrifugation and repeated washing with PBS

buffer. The amino-modified cyanobacteria were collected by centrifugation and washed with PBS for three times.

2. Upconversion cyanobacteria (UCCy)

5 mg of Cit-UCNPs was added to PBS solution of amino-modified cyanobacteria (10^6 cells mL^{-1}, 10 mL), then 1-(3-Dimethylaminopropyl)-3-ethylcarbodiimide hydrochloride (EDC·HCl, 10 mg) was added. The reaction mixture was allowed standing for 2 h. The UCCy was collected by centrifugation and washed with PBS for three times to remove free Cit-UCNPs.

3. UCCy@Gel

UCCy@Gel was prepared by the free-radical polymerization at the surface of UCCy. Briefly, in the preservation of argon, UCCy (1×10^6 cells mL^{-1}) was added into a PBS buffer solution of DMAPMA, NMS, MA and GDA at a weight ratio of 1.2 :0.6 :1 :2, and the polymerization was initiated by the addition of APS (0.6 mg mL^{-1}) and TEMED (0.6 μL mL^{-1}) and performed at 25 °C for 60 min to produce hydrogel encapsulated UCCy. Finally, hydrogel coated UCCy was reacted with 4-aminophenylboronic acid hydrochloride to obtain UCCy@Gel.

Cell culture

High-glucose Dulbecco's Modified Eagle Medium (DMEM Gibco, USA) and Roswell Park Memorial Institute 1640 (RPMI 1640, Gibco, USA) were used to culture rat cardiomyocytes (H9C2 cells), mouse macrophage cell line (RAW 264.7), human umbilical vein endothelial cells (HUVECs) and mouse fibroblast cells (L929). The cell culture medium contained 10% heat-inactivated fetal bovine serum (FBS), streptomycin (100 mg mL^{-1}) and penicillin (100 mg mL^{-1}). All cell lines were purchased from Shanghai Institute of Cell Medicine, Chinese Academy of Sciences and cultured in a cell incubator at 37 °C and 5% carbon dioxide.

Synechococcus elongatus PCC 7942 was purchased from the Freshwater Algae Culture Collection at the Institute of Hydrobiology (FACHB-805; Chinese Academy of Sciences, Wuhan, China). The cyanobacteria were incubated in 250 mL flasks containing BG-11 liquid medium in a 28 °C incubator, illuminated by a 50 W plant fluorescent bulb (Philips Co., Ltd., China). Fresh BG-11 medium was added daily to the culture to maintain a constant volume. The cyanobacteria were maintained with fixed diurnal cycle which was a 12 h light and 12 h dark cycle. Every 4 days, approximately 25% of the cyanobacteria were discarded and replaced with an equal volume of fresh

BG-11 medium.[2]

CCK-8 cytotoxicity assay

H9C2, RAW 264.7, HUVECs and L929 cells were plated in 96-well plates at a density of 1×10^4 cells per well and cultured overnight. After the cells adhered to the wall and grew normally, UCCy@Gel with different concentrations in the range of 10^5 to 10^7 cells mL^{-1} were added to the culture plates. Subsequently, 10 μL cell counting kit-8 (CCK-8) was added to the culture system and the optical density (OD) was measured with a microplate reader (Synergy 2, Bio-TEK) at a reference wavelength of 450 nm after 2 h incubation. Cell viability was calculated as follows (A represents the value of optical density):

$$Cell\ viability = \frac{[A\ (experimental\ well) - A\ (blank\ well)]}{[A\ (control\ well) -\ A\ (blank\ well)]} \times\ 100\% \#(1)$$

Calcein-acetoxymethyl ester (AM)/propidium iodide (PI) double staining method

H9C2, L929, HUVECs and RAW 264.7 cells were seeded in a 24-well plate with 2×10^4 cells per well and cultured at 37 °C and 5% CO_2. After the cell growth was stable, the different concentrations of UCCy@Gel ($1 \times 10^4 - 1 \times 10^7$ cells mL^{-1}) were co-cultured with the cells for 24 h. After that, the medium was removed and rinsed with sterile PBS. Then 2 μM calcein-AM and 8 μM PI were added to incubate for 45 min. Finally, the cells were washed with PBS and observed using an inverted fluorescence microscope.

Cell surface immunofluorescence staining

Cells were fixed with 4% paraformaldehyde for 10 min, following by a wash with ice-cold PBS for three times. After fixation, cells were permeabilized with 0.2% Triton X-100 in PBS followed by blocking with goat serum. Primary antibodies (Anti-CD86 and Anti-CD206, Abcam, Shanghai) were incubated overnight at 4 °C. The next day, samples were incubated with secondary antibodies for 2 h at room temperature and protected from light. The nuclei were then counterstained with 4',6-diamidino-2-phenylindole (DAPI). Results were analyzed by using a fluorescence microscope.

Hydrogen peroxide-induced cell model

The operations were performed following a reported method.[3] H9C2 cells were cultured in DMEM medium with 10% FBS. The cells were passaged every 2 days. Cells were seeded into 96-well plates at a density of 6×10^3 cells per well within log-growth phase. The injury model of mice cardiomyocytes was established by 2 h

treatment of H9C2 cells with freshly prepared culture medium containing H_2O_2 (400 μM). The cardiomyocytes were divided into 5 groups: blank control (without any treatment), model (incubation with culture medium containing H_2O_2 (400 μM) without FBS for 2 h), UCCy@Gel ＋NIR group (incubation with UCCy@Gel (1×10^6 cells mL^{-1}) and illumination with 980 nm NIR light), UCCy@Gel group (incubation with UCCy@Gel (1×10^6 cells mL^{-1}) and NIR group (illumination with 980 nm NIR light). The H_2O_2 working solution was added and incubated with the cells for 2 h after that the cardiomyocytes were treated for 12 h. Cells were collected for use in subsequent experiments.

Polarization of macrophages

The operations were performed following a reported method.[4] M0 macrophages were supplied with the high-glucose DMEM medium containing FBS (10%) in an incubator at 37 °C with 5% CO_2. Cells were digested with 0.25% trypsin and passaged when cells confluency reached 80%. Cells were seeded into 12-well plates at a density of 1×10^5 cells per well within log-growth phase. After overnight culture, adherent cells were selected and stimulated with lipopolysaccharide (LPS, 50 ng mL^{-1}) for 24 h to obtain M1 macrophages. The macrophages were then divided to 5 groups: Control (M0 macrophages), model (LPS treatment, 50ng mL^{-1}), UCCy@Gel ＋NIR group, UCCy@Gel group and NIR group. Cells were treated for 12 h and collected for use in subsequent experiments.

ELISA

For cytokine analysis, cell culture supernatants and tissue homogenates were centrifuged (12,000 rpm for 12 min) and supernatants were collected and stored at −80 °C. After preparation of samples, reactants and standards following the above steps, the mixture was let react at 37 °C for 90 min. After plates were washed, biotinylated antibody working solutions (IL-4, IL-10, IL-6 and TNF-α, Camilo Bio-Engineering Co Ltd, Nanjing) were added, and plates were incubated at 37 °C for 60 min. Then, ABC working solution and TMB standard solution were added, respectively. Inflammatory factors quantity was determined according to a standard curve of samples.

Animals

Male C57BL/6 mice (9−10 weeks old) were initially purchased from the Model Animal Research Center of Nanjing University and bred in the animal facility of Nanchang University. The mice were housed 3−5 mice per cage with and libitum

access to food and water, and were maintained in a room (22 ± 2 °C) under a 12 h light and 12 h dark cycle (light on at 07:00 a.m. and off at 07:00 p.m.). All animal experiments were performed under the guidelines of the National Institutes of Health and approved by the Animal Ethics Committee of Nanchang University, Nanchang, China (SYXK 2018-0006).

Myocardial infarction (MI) model

The mice were anesthetized by intraperitoneal injection, intubated and placed on a rodent ventilator. The thorax was opened at the left fourth intercostal space and the left anterior descending coronary artery (LAD) was ligated with 7−0 prolene suture. The blank group procedure was performed identically without the ligation but moved it behind the LAD artery. The model was proven to be successful when the anterior wall of the left ventricle turned pale and there were clinical manifestations of acute myocardial infarction.[5]

Prevention and treatment of mice myocardial infarction (MI) model

Under small animal ultrasound guidance, the UCCy@Gel (10 μL) was injected into the endomyocardial using a micro-syringe. Following surgery, the mice were kept in a dark environment for 24 h. Afterward, the mice MI model were inducted as previously described. After the vital signs were stabilized, the hearts of mice were irradiated with 980 nm NIR once daily for 2 h. After 3 days, mice were euthanized by CO_2 asphyxiation, and hearts were collected for subsequent treatment effect verification.

Transthoracic echocardiography

A Vevo 2100 high-revolution imaging system with 30 MHz sensor was used to measure echocardiographic parameters to assess left ventricular function of mice. At every point time, left ventricular internal diameter (LVIDd) and left ventricular systolic internal diameter (LVIDs) were obtained from a parasternal short-axis view and an apical M-shaped trace. The corresponding formulas were used to calculate the percentages of left ventricular ejection fraction (LVEF) and left ventricular ejection fraction shortening (LVFS).

Histological analysis

After echocardiographic detection, the heart tissues from mice were fixed in paraformaldehyde for 24 h, dehydrated by increasing the concentration of ethanol and then encased in paraffin. Masson trichrome staining was performed on 5 μm heart

tissue sections. Left ventricular scar circumference percentage was quantified by ImageJ analysis software. Triphenyl tetrazole chloride (TTC) staining was used to determine the size of the myocardial infarction, as described in the literature.[6]

Hematoxylin-eosin (H&E) staining, Masson staining and Giemsa staining

The tissue of heart was fixed with 10% formaldehyde and embedded with paraffin. After 5 weeks decalcification, paraffin sections (4 μm) were made on the microtome. Then H&E staining and light microscope were used to observe the pathological changes of the joint. Then masson staining and optical microscope were used to observe the degree of joint fibrosis. At last, the degree of apoptosis in the joint was observed under light microscope.

Triphenyltetrazolium chloride (TTC) staining

First, the isolated heart was placed on a pathological glass slide, placed flat in dry ice, frozen for 10 min, taken out and sliced, and the whole heart was cut into 5 slices on average. Subsequently, all sections were placed in 2% triphenyltetrazolium chloride phosphate buffer (pH 7.4) in a 37°C water bath for 3 min. (protect from light, oscillate). Finally, the samples were photographed after staining and stored in 10% formalin.

Immunohistochemistry and immunofluorescence

For immunohistochemical staining, the paraffin sections were immersed in methanol with 3% H_2O_2 for 25 min at room temperature to reduce endogenous peroxidase activity and dewaxed by xylene and gradient ethanol in water. Then, sections were immersed in antigen repair buffer (sodium citrate, pH 6.0) and heated in a microwave. After natural cooling, the sections were placed in PBS (pH 7.4) and shaken on the decolorizing shaker 3 times for 5 min each time. Sections were blocked using 3% BSA for 30 min and then labeled with primary antibodies followed by secondary antibodies. Furthermore, the sections were washed with tap water to terminate the chromogenic reaction, counter-stained with hematoxylin. Finally, after dehydration with gradient ethanol and permeabilization with xylene, the sections were sealed.

For immunofluorescence, the paraffin sections were dewaxed by xylene and gradient ethanol in water. Then, sections were immersed in antigen repair buffer (Ethylenediaminetetraacetic acid disodium salt, EDTA, pH 8.0) and heated in a microwave. After natural cooling, the sections were placed in PBS (pH 7.4) and shaken on the decolorizing shaker 3 times for 5 min each time. Sections were blocked using 3% BSA for 30 min and then labeled with primary antibodies followed by secondary

antibodies. In addition, DAPI was used to redye nuclei, and autofluorescence was quenched with autofluorescence quencher. Finally, the anti-fluorescence quencher was used to seal the sections.

Triphenyltetrazolium chloride (TTC) staining

First, the isolated heart was placed on a pathological glass slide, placed flat in dry ice, frozen for 10 min, taken out and sliced, and the whole heart was cut into 5 slices on average. Subsequently, all sections were placed in 2% triphenyltetrazolium chloride phosphate buffer (pH 7.4) in a 37°C water bath for 3 min. (protect from light, oscillate). Finally, the samples were photographed after staining and stored in 10% formalin.

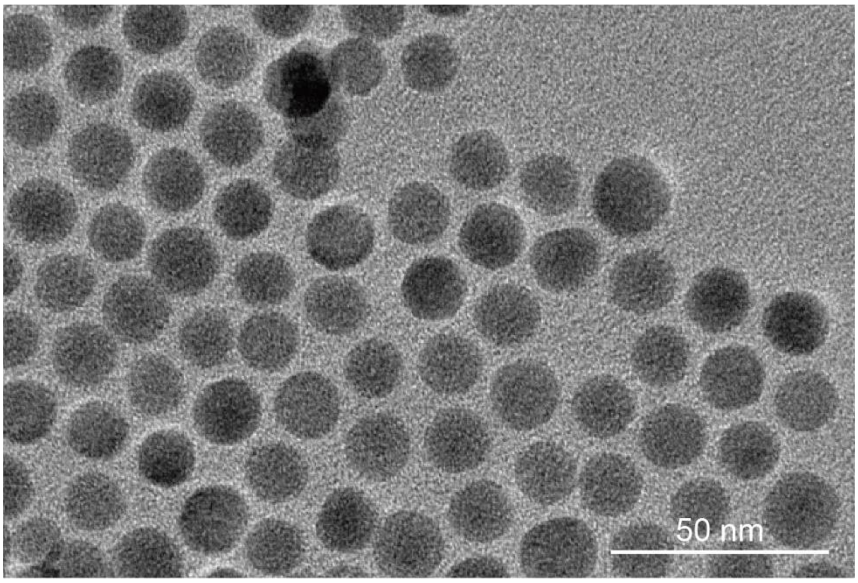

Figure S1. The TEM image of β-$NaErF_4$ core. Scale bar = 50 nm.

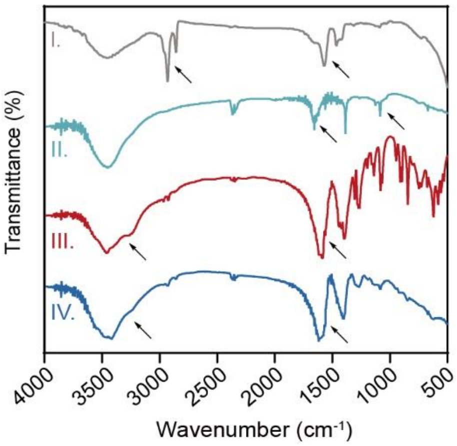

Figure S2. The FTIR spectra of I-OA-UCNPs; II-UCNP(BF_4); III-Sodium citrate; IV-Cit-UCNPs. Characteristic peaks: I-2936 cm^{-1}, alkyl C-H stretching; 1579 cm^{-1}, C=C stretching; II-1654 cm^{-1}, C=O stretching; 1083 cm^{-1}, BF_4^-; III-3452 cm^{-1}, C-O stretching; 1617 cm^{-1}, C=O stretching; 3416 cm^{-1}, C-O stretching; 1626 cm^{-1}, C=O stretching.

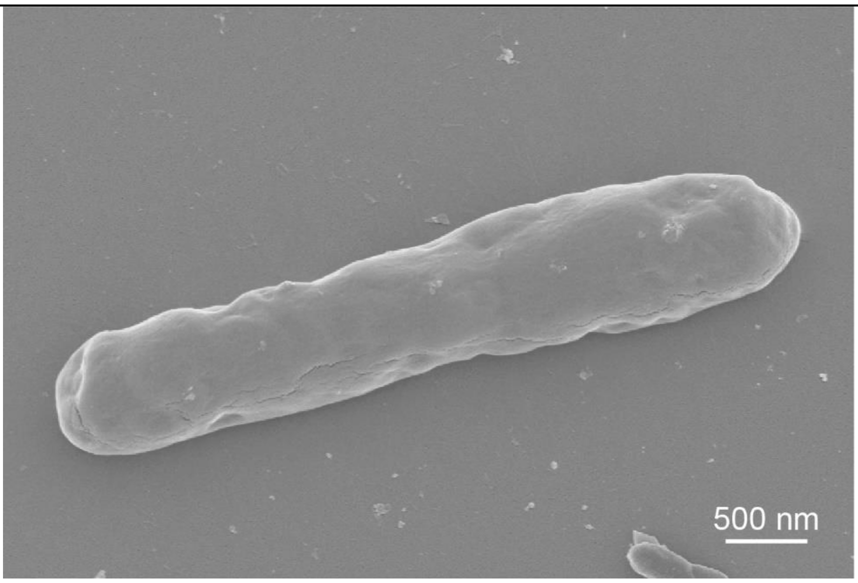

Figure S3. The SEM image of cyanobacteria. Scale bar = 500 nm.

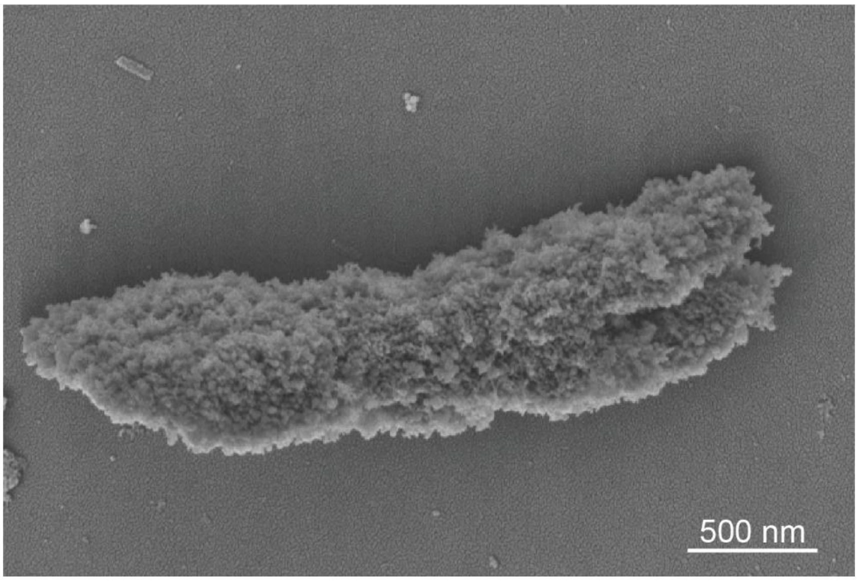

Figure S4. The SEM image of UCCy. Scale bar = 500 nm.

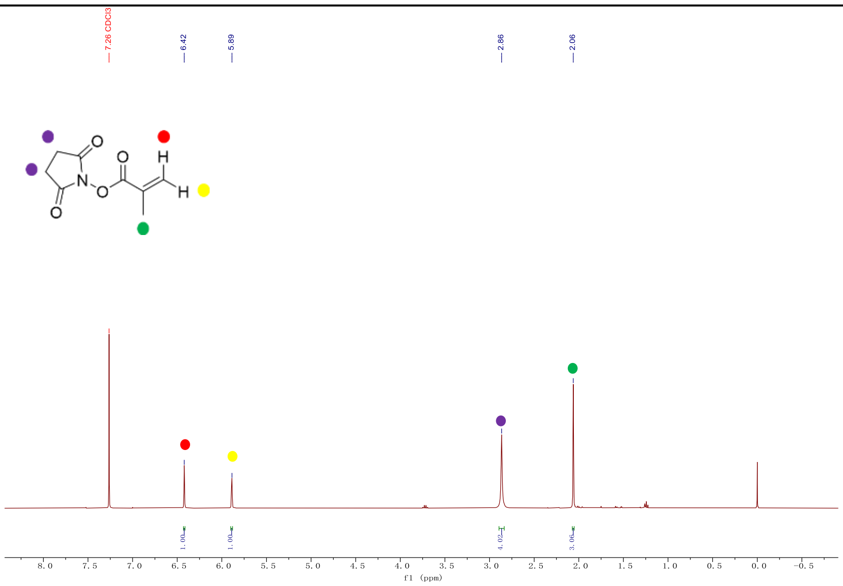

Figure S5. The ^{1}H NMR spectrum of N-succinimidyl methacrylate in $CDCl_3$.

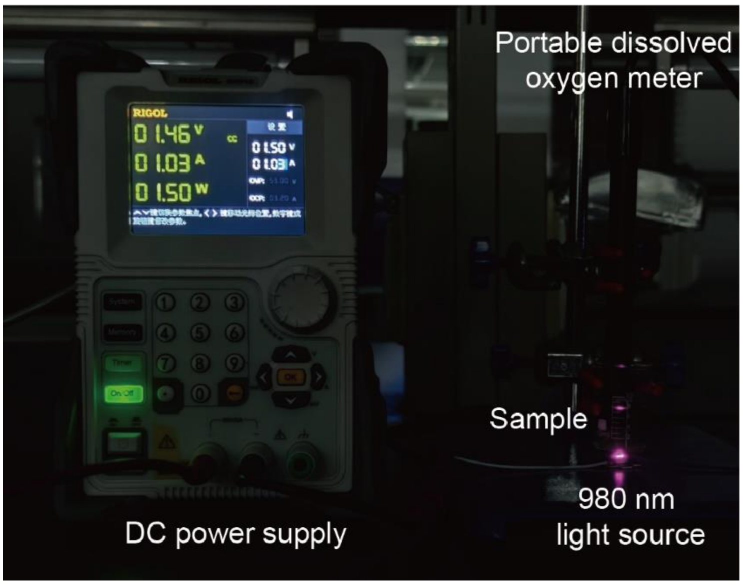

Figure S6. The experiment devices of dissolved oxygen measurement.

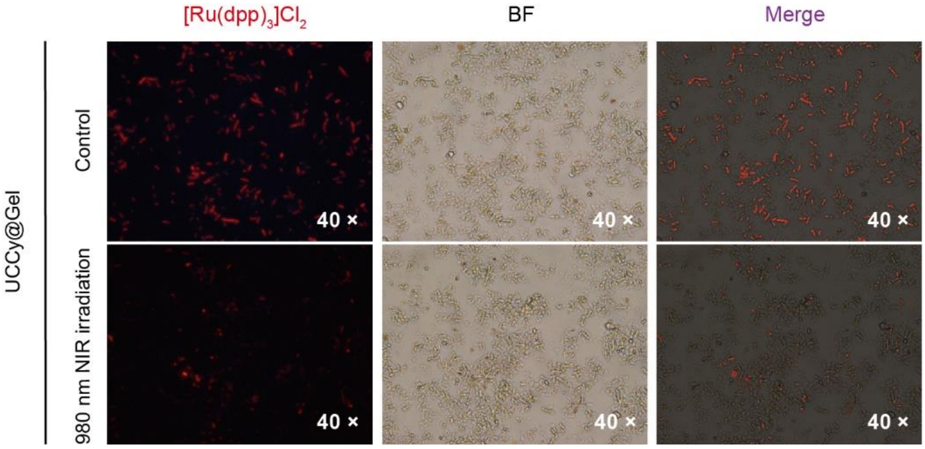

Figure S7. The oxygen liberation inside UCCy@Gel was monitored by $Ru(dpp)_3Cl_2$ probe. Scale bar = 40 ×.

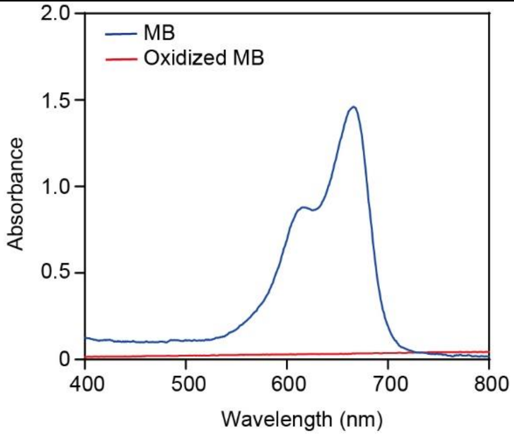

Figure S8. The UV–vis spectra of MB and MB + Fenton's reagent.

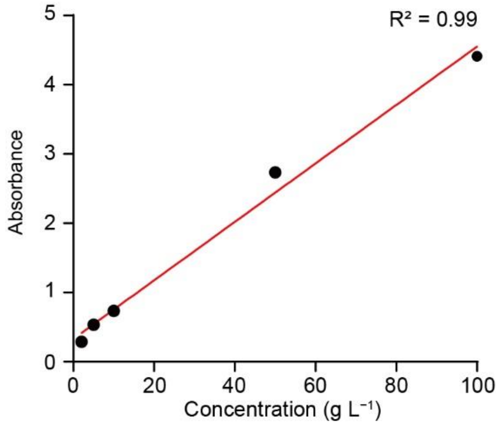

Figure S9. The standard curve of MB.

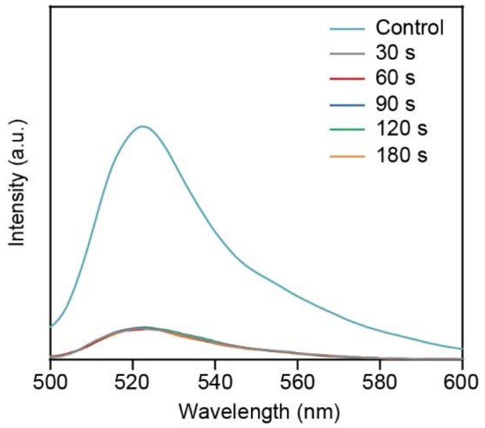

Figure S10. The fluorescence spectra of dichlorodihydrofluorescein (DCFH) probe in Fenton's reagent with UCCy@Gel.

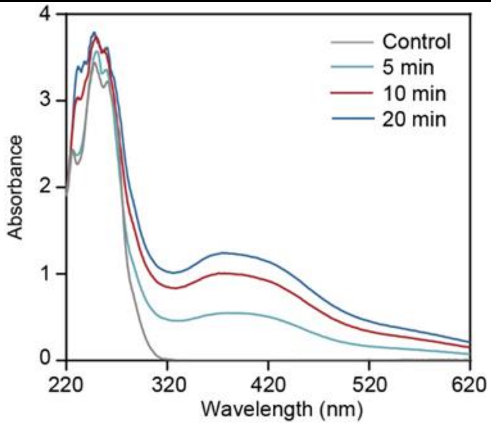

Figure S11. The UV–vis spectra of 4-aminophenylboronic acid hydrochloride treated with hydrogen peroxide.

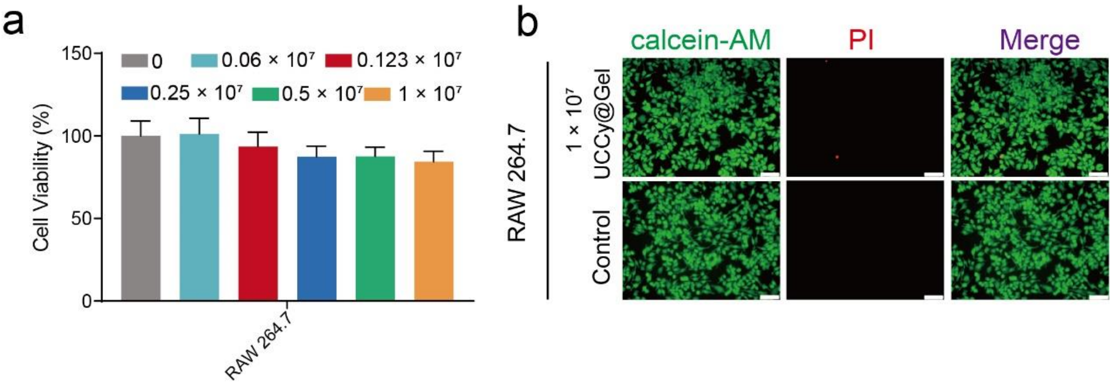

Figure S12. a) Cell viability and b) live/dead staining of RAW 264.7 cocultured with different concentrations of UCCy@Gel. Scale bar = 100 μm.

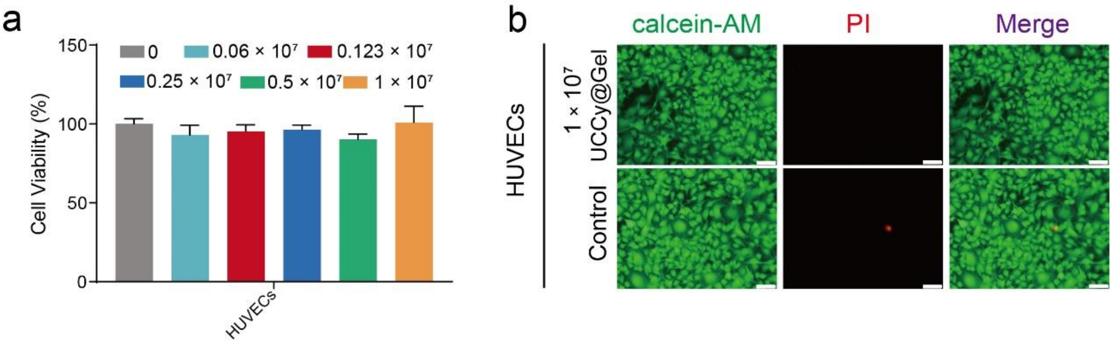

Figure S13. a) Cell viability and b) live/dead staining of HUVECs cocultured with different concentrations of UCCy@Gel. Scale bar = 100 μm.

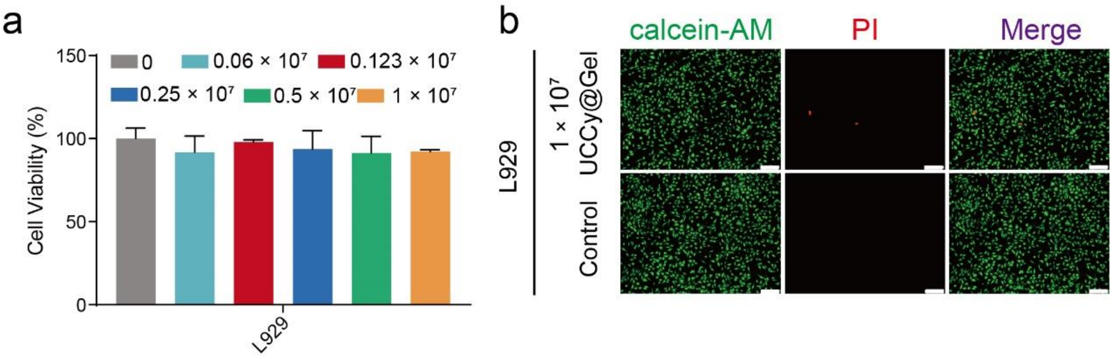

Figure S14. a) Cell viability and b) live/dead staining of L929 cocultured with different concentrations of UCCy@Gel. Scale bar = 100 μm.

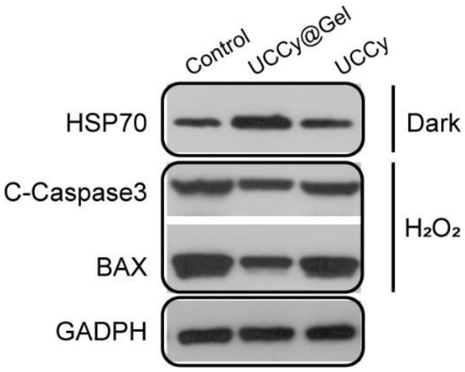

Figure S15. The expression of HSP70, C-Caspase-3 and BAX of H9C2 cells with different treatments.

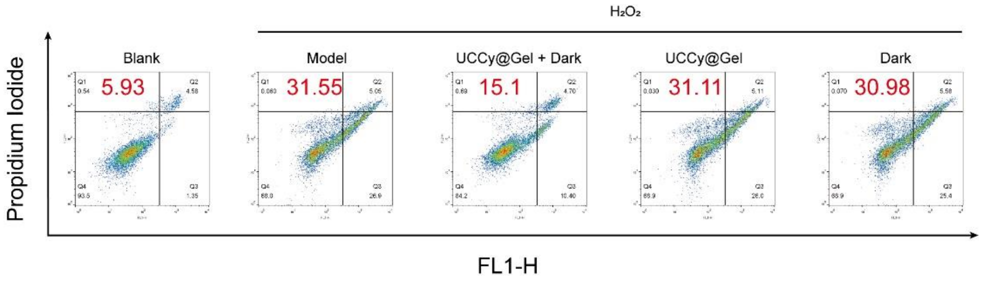

Figure S16. The apoptosis rate of each group detected by flow cytometry.

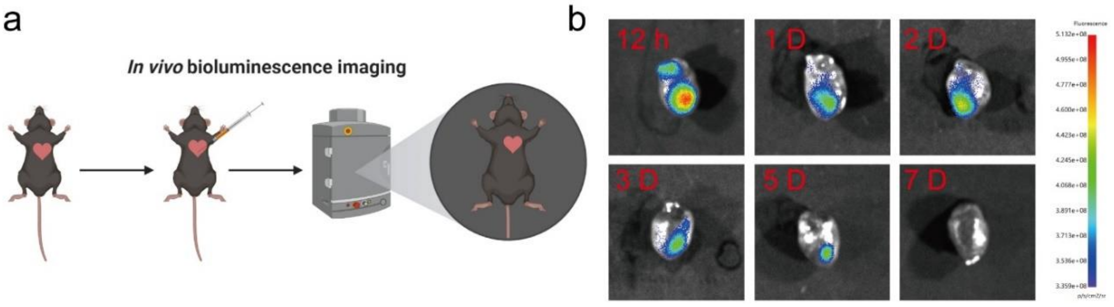

Figure S17. Change of fluorescence (1 D-7 D) with small animal imaging system after the apical injection of UCCy@Gel.

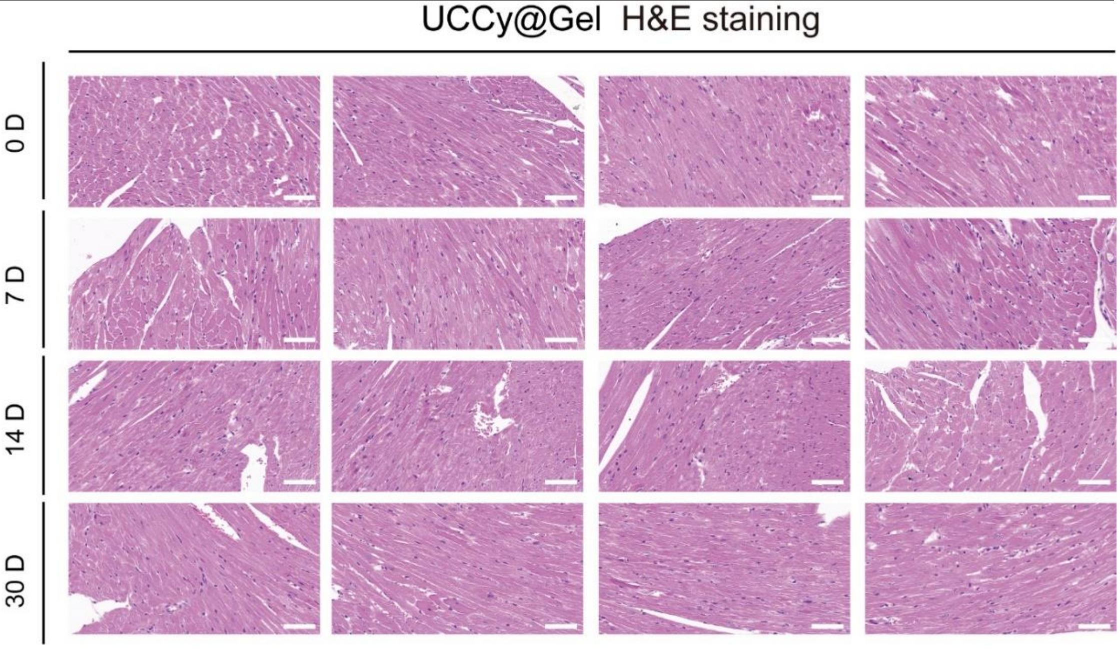

Figure S18. The safety of the UCCy@Gel was further studied *in vivo* (0 D-30 D). Scale bar = 100 μm.

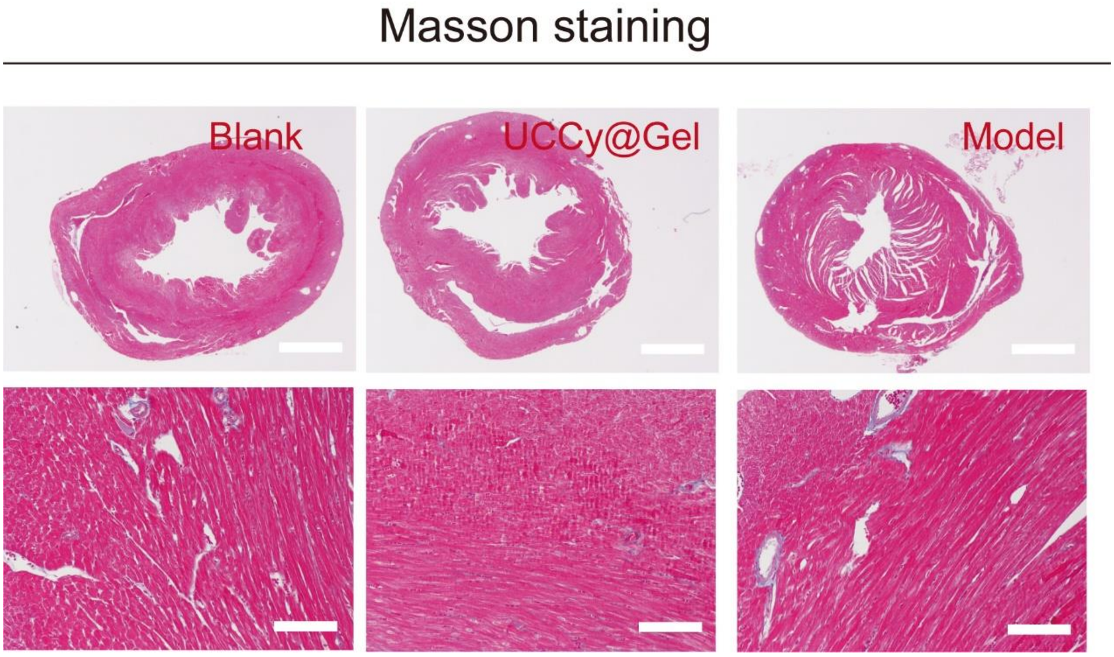

Figure S19. The Masson staining of mice hearts demonstrated protection effect. Scale bar = 1 mm. Scale bar = 100 μm.

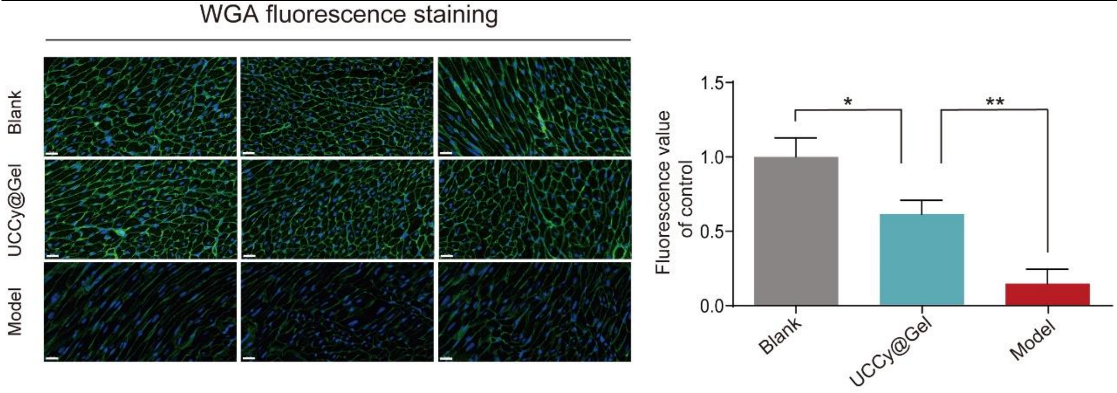

Figure S20. WGA fluorescence staining of MI mice with different treatments after 6 h. Scale bar = 20 μm.

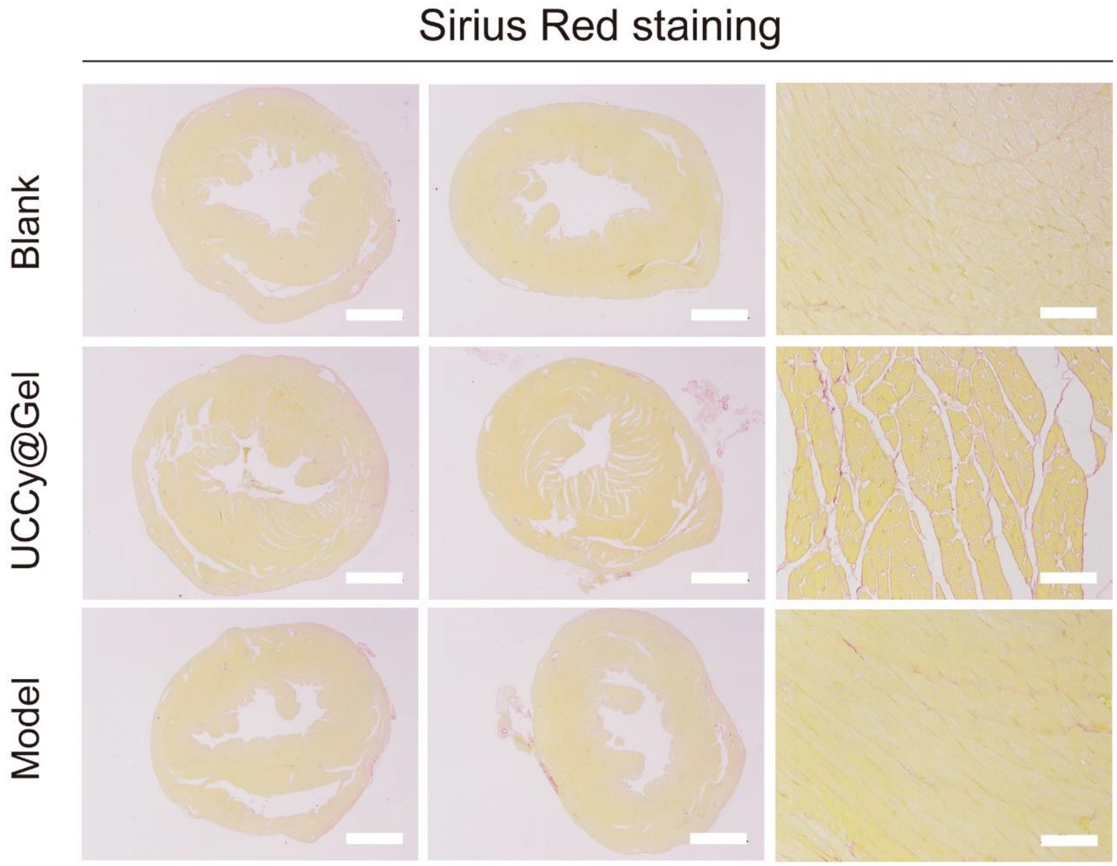

Figure S21. Sirius Red staining of MI mice with different treatments after 6 h. Scale bar = 1 mm. Scale bar = 50 μm.

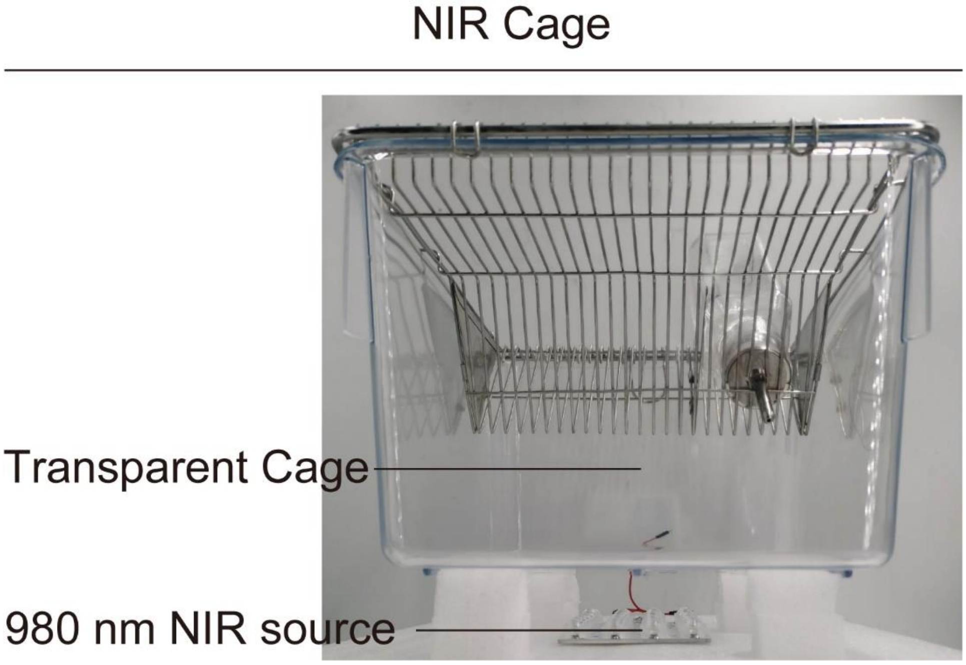

Figure S22. The NIR treatment cage.

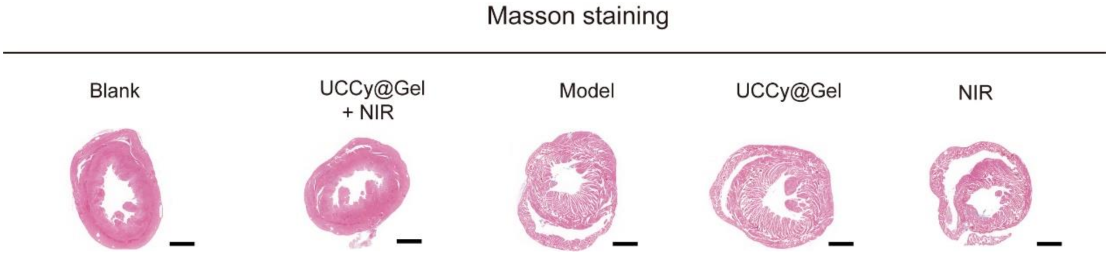

Figure S23. Masson staining of heart collected from each group mice after the treatments. Scale bar = 1 mm.

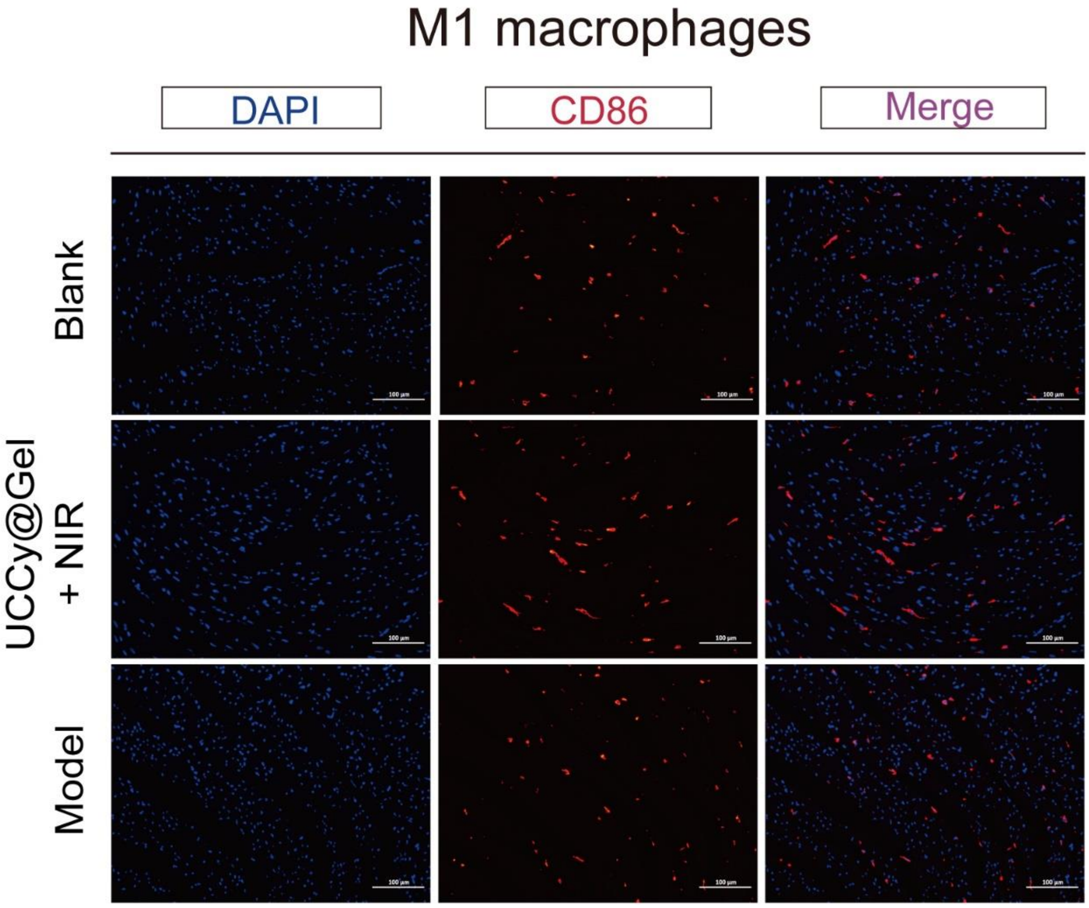

Figure S24. CD86 staining of heart collected from each group mice after the treatment. Scale bar = 100 μm.

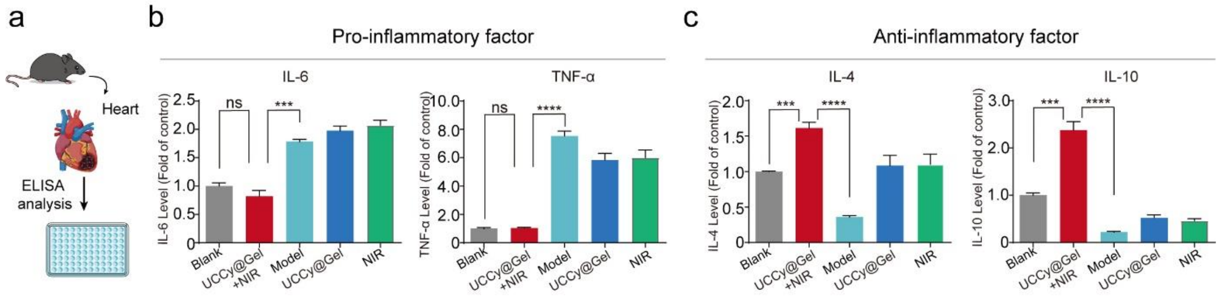

Figure S25. a) Schematic diagram of inflammatory factors in heart tissues. b) ELISA results showed that UCCy@Gel prevented MI-induced increase of pro-inflammatory factors including IL-6 (left panel) and TNF-α (right panel). c) ELISA results showed that UCCy@Gel reversed MI-induced decrease of anti-inflammatory factors including IL-4 (left panel) and IL-10 (right panel).

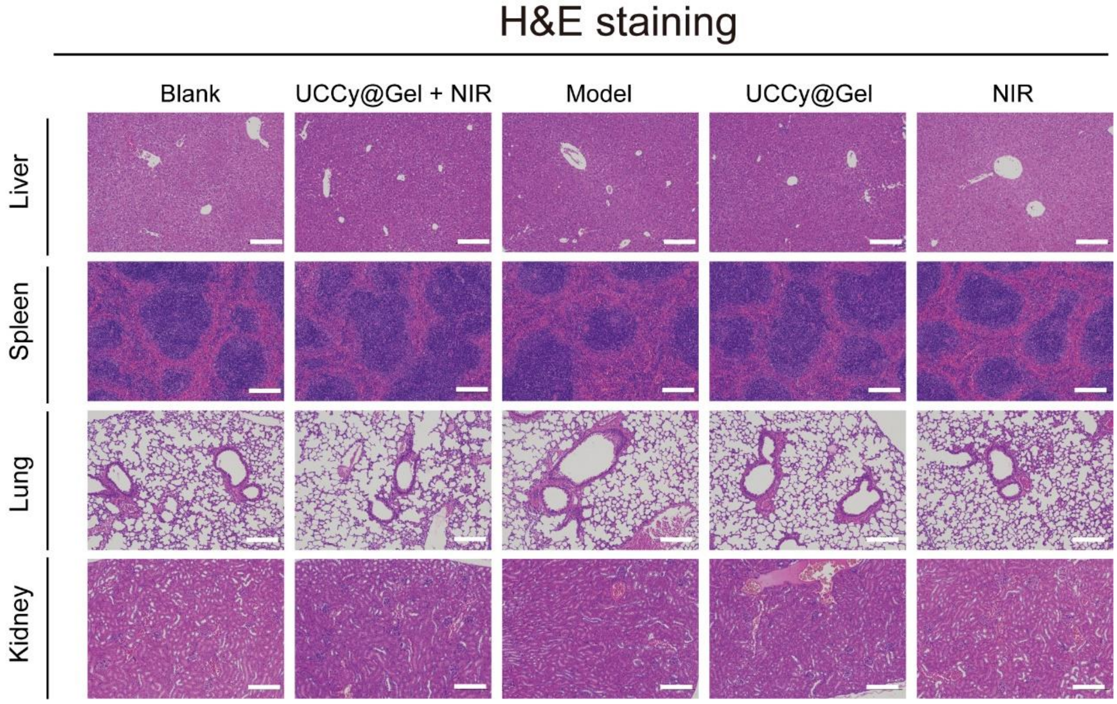

Figure S26. H&E staining of the important organs (liver, spleen, lung and kidney) collected from each group mice after the treatments. Scale bar = 200 μm.

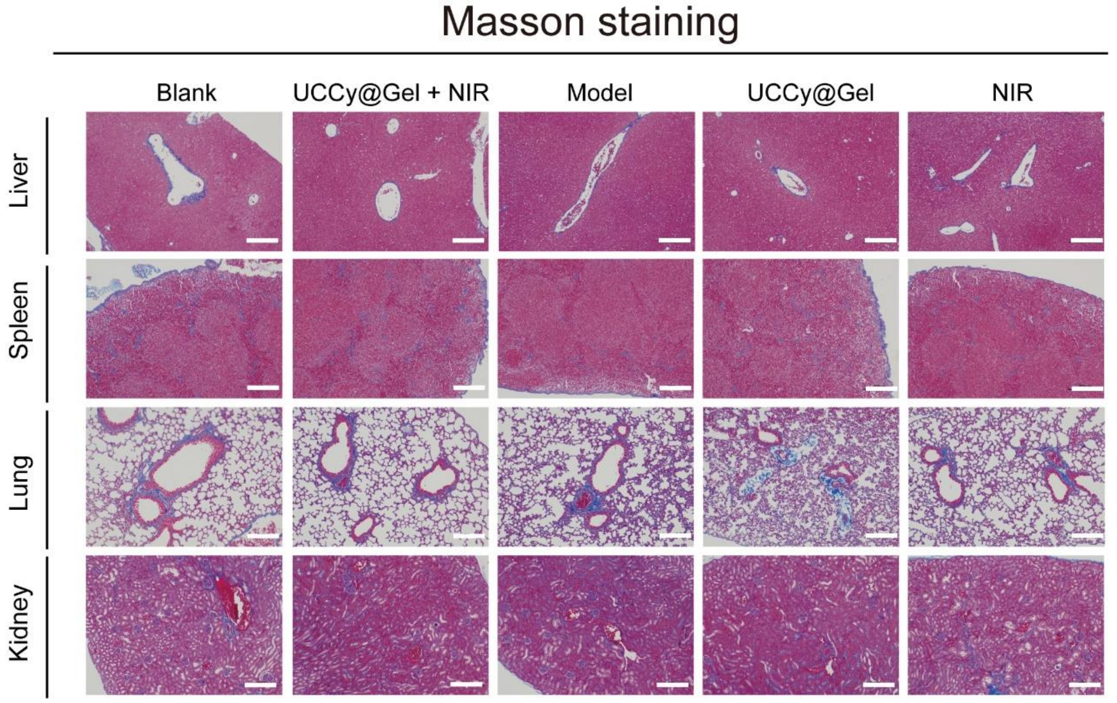

Figure S27. Masson staining of the important organs (liver, spleen, lung and kidney) collected from each group mice after the treatments. Scale bar = 200 μm.

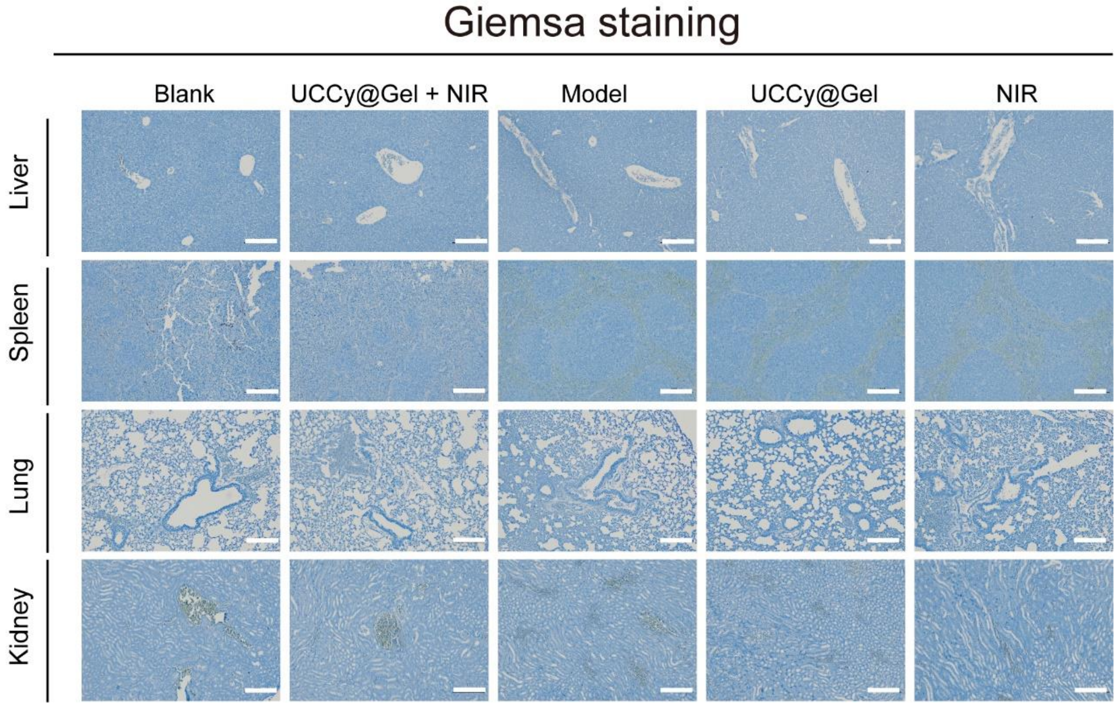

Figure S28. Giemsa staining of the important organs (liver, spleen, lung and kidney) collected from each group mice after the treatments. Scale bar = 200 μm.

References for supporting information

[1] A. Dong, X. Ye, J. Chen, Y. Kang, T. Gordon, J. M. Kikkawa, C. B. Murray, *J. Am. Chem. Soc.* **2011**, *133*, 998.
[2] E. Suzuki, K. Umeda, S. Nihei, K. Moriya, H. Ohkawa, S. Fujiwara, M. Tsuzuki, Y. Nakamura, *Biochim. Biophys. Acta* **2007**, *1770*, 763.
[3] C. H. Law, J. M. Li, H. C. Chou, Y. H. Chen, H. L. Chan, *Toxicology* **2013**, *303*, 54.
[4] M. C. Schmid, S. Q. Khan, M. M. Kaneda, P. Pathria, R. Shepard, T. L. Louis, S. Anand, G. Woo, C. Leem, M. H. Faridi, T. Geraghty, A. Rajagopalan, S. Gupta, M. Ahmed, R. I. Vazquez-Padron, D. A. Cheresh, V. Gupta, J. A. Varner, *Nat. Commun.* **2018**, *9*, 5379.
[5] J. E. Cohen, A. B. Goldstone, M. J. Paulsen, Y. Shudo, A. N. Steele, B. B. Edwards, J. B. Patel, J. W. MacArthur, M. S. Hopkins, C. E. Burnett, K. J. Jaatinen, A. D. Thakore, J. M. Farry, V. N. Truong, A. T. Bourdillon, L. M. Stapleton, A. Eskandari, A. S. Fairman, W. Hiesinger, T. V. Esipova, W. L. Patrick, K. Ji, J. A. Shizuru, Y. J. Woo, *Sci. Adv.* **2017**, *3*, 37.
[6] M. C. Fishbein, S. Meerbaum, J. Rit, U. Lando, K. Kanmatsuse, J. C. Mercier, E. Corday, W. Ganz, *Am. Heart J.* **1981**, *101*, 593.
